# Efficacy of various acupuncture modalities on alleviating symptoms in Parkinson’s disease: a systematic review and meta-analysis of randomized controlled trials

**DOI:** 10.1007/s10072-025-08333-1

**Published:** 2025-07-17

**Authors:** Peihao Yu, Guiqian Wang, Sanchun Tan, Mingming Zhan, Yiwei Zheng, Wei Zhou, Hanzhang Li, Jun Ma

**Affiliations:** 1 College of Acupuncture-Moxibustion and Orthopaedics, Hubei University of Chinese Medicine, Wuhan, 430065 China; 2 Hubei Shizhen Laboratory, Wuhan, 430061 China; 3 Hubei Provincial Chinese Hospital, Wuhan, 430061 China

**Keywords:** Parkinson’s disease, Acupuncture, Motor function, Sleep, Anxiety

## Abstract

**Background:**

Parkinson’s disease (PD) is a progressive neurological condition, that often respond poorly to conventional treatments. Acupuncture has gained attention as a supportive therapy, but the clinical effects of its various modalities remain insufficiently defined. This study examined the therapeutic efficacy of different acupuncture approaches in managing symptoms of PD.

**Methods:**

The meta-analysis and systematic review was carried out in accordance with PRISMA 2020 guidelines and registered in PROSPERO (CRD42024627483). Randomized controlled trials (RCTs) assessing acupuncture modalities combined with standard PD therapy was encompassed. The Cochrane RoB 2 tool was utilized to figure out the risk of bias, and subgroup/meta-regression analyses explored heterogeneity. Evidence certainty was rated using the GRADE framework.

**Results:**

In 50 RCTs (n = 3,248), acupuncture significantly outperformed Western medicine across all UPDRS domains, with the strongest effect on treatment-related motor complications (SMD: –2.16; 95% CI: –3.10 to –1.22). It also improved quality of life, pain, sleep, depression, and anxiety. Optimal results were linked to ≥ 10 acupoints, moderate session duration, and thrice-weekly treatment. Among all modalities, electroacupuncture showed the highest overall efficacy. Thinner needles worked better for non-motor symptoms; thicker ones favored motor outcomes. No serious adverse events occurred; mild effects were rare and transient.

**Conclusions:**

This comprehensive analysis highlights acupuncture—especially electroacupuncture—as a clinically valuable adjunct to standard PD therapy. Its benefits across motor, non-motor, and psychological domains, when delivered with optimized parameters, suggest a strong case for its integration into personalized PD management strategies.

**Graphical Abstract:**

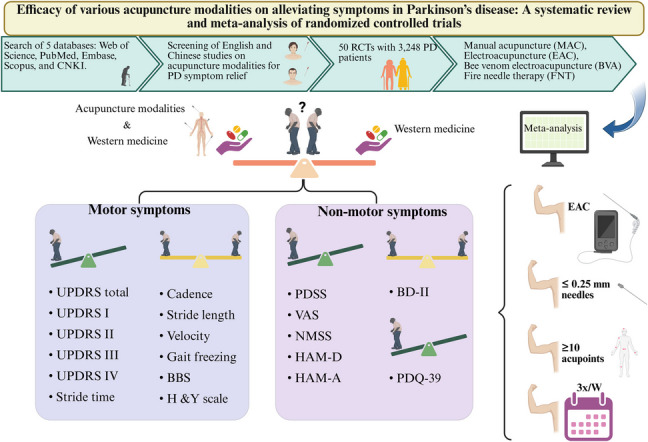

**Supplementary Information:**

The online version contains supplementary material available at 10.1007/s10072-025-08333-1.

## Introduction

Parkinson’s disease (PD) is a convoluted neurodegenerative condition defined by gradual dopaminergic nerve loss, resulting in a wide range of motor and non-motor symptoms [1, 2]. While pharmacological interventions, including levodopa as well as agonists of dopamine, continue to serve as the foundation for managing PD, their long-term use is associated with diminishing efficacy and significant side effects, including motor fluctuations, dyskinesia, and non-motor complications [3, 4]. Surgical interventions, such as deep brain stimulation (DBS), offer alternative treatment options, yet they are invasive, expensive, and not universally applicable [5, 6]. Given these challenges, there is increasing interest in complementary and integrative medicine (CIM), particularly acupuncture, as an adjunctive therapy for PD [7, 8]. Acupuncture, a fundamental component of traditional Chinese medicine (TCM), involves the insertion of fine needles into specific acupoints to restore physiological balance. Major modalities include manual acupuncture (MAC), electroacupuncture (EAC) with electrical current, bee venom acupuncture (BVAC) using diluted venom, and fire needle therapy (FNT), which combines mechanical and thermal stimulation via heated needles [9, 10]. Owing to its neuroprotective and neuromodulatory potential, acupuncture has been extensively investigated for its effects on both motor and non-motor symptoms of the disease [11, 12]. Despite a growing body of research, existing systematic reviews and meta-analyses on acupuncture for PD have several critical limitations. Many studies focus on single acupuncture modalities without systematically considering the relative efficacy of different techniques [13, 14]. Furthermore, methodological inconsistencies— encompassing differences in approaches to therapy and measures of outcome, as well as limitations such as limited sample sizes and insufficient control conditions—complicate efforts to draw definitive conclusions [13–17]. While some meta-analyses have suggested potential benefits of acupuncture for symptom management, the heterogeneity in study design and the absence of high-quality randomized controlled trials (RCTs) with robust methodologies weaken the generalizability of these findings [13, 15–18]. Additionally, safety assessments in previous studies have often been inadequate, leaving uncertainties regarding the adverse effects and long-term risks of acupuncture interventions [13, 16, 17]. Moreover, current research has largely focused on improvements in motor function, with less emphasis on non-motor symptoms and broader clinical outcomes that impact patient quality of life [14, 15, 17–19]. Critical measures such as disease severity staging, functional mobility parameters, gait performance, cognitive and emotional well-being, and overall quality of life remain underexplored in comparative analyses [13, 14, 17, 18]. Existing studies rarely integrate comprehensive assessments that encompass both motor and non-motor domains, limiting their applicability in clinical decision-making [13, 16–18]. Furthermore, few studies have systematically examined changes in validated scales assessing disease severity, mobility, psychiatric symptoms, and daily life impact, making it difficult to determine the full therapeutic scope of acupuncture in PD management [13, 15–18]. To address these gaps, this research undertakes a comprehensive review and meta-analysis to gauge the effectiveness of various acupuncture modalities across multiple PD-related outcomes. The findings aim to clarify acupuncture’s role in managing motor, non-motor, and quality-of-life domains, offering evidence to guide clinical practice and integrative care approaches.

## Methods

This research has been duly registered with the International Prospective Register of Systematic Reviews (PROSPERO) under the identifier CRD42024627483 and strictly follows the Preferred Reporting Items for Systematic Reviews and Meta-Analyses (PRISMA 2020) guidelines [20].

### Search strategy

A thorough investigation was carried out across Web of Science, PubMed, Embase, Scopus, and China National Knowledge Infrastructure (CNKI)—covering the period from January 1, 2000, to September 30, 2024. Medical Subject Headings (MeSH) terms related to “*Parkinson’s Disease,*” “*Acupuncture*” and “*Acupuncture Modalities,*” were utilized. To identify other related publications, the bibliographical lists of the selected studies were examined.

### Eligibility principles

#### Inclusion criteria

This study included individuals diagnosed with idiopathic PD, with no restrictions on age, sex, race, disease severity, or disease duration. Included studies specifically enrolled participants without comorbid neurological or movement disorders. Each patient in the intervention group received one specific type of acupuncture—MAC, EAC, BVAC, or FNT—administered in combination with Western medicine (WM). In contrast, patients in the control group received WM alone. Standard treatments consisted of a combination of L-dopa, dopamine agonists, and other pharmacological agents. Only prospective RCTs published in full-text format and in English and Chinese were considered eligible. The primary outcome required the inclusion of at least one version of the Unified Parkinson’s Disease Rating Scale (UPDRS), encompassing either the total score or subscales I–IV. Secondary outcomes included: 1) quality of life, assessed using the Parkinson’s Disease Questionnaire-39 (PDQ-39); 2) sleep disturbances, measured by the Parkinson’s Disease Sleep Scale (PDSS); 3) non-motor symptoms, evaluated using the Non-Motor Symptoms Scale (NMSS); 4) pain, assessed via the Visual Analog Scale (VAS); 5) mood disorders, evaluated with the Bipolar Disorder-II Scale (BD-II), the Hamilton Depression Rating Scale (HAM-D), and the Hamilton Anxiety Rating Scale (HAM-A); 6) disease stage, measured using the Hoehn and Yahr scale; and 7) gait and balance parameters—including cadence, stride length, walking speed, stride time, and freezing episodes—measured via the GAITRite system, with balance further assessed using the Berg Balance Scale (BBS).

#### Exclusion criteria

1) studies unrelated to PD; 2) non-RCTs; 3) unpublished or non–full-text reports; 4) absence of a defined primary outcome; and 5) interventions not matching the comparison of one acupuncture modality plus WM versus WM alone.

#### Data extraction

Three reviewers individually checked the titles and abstracts (P.Y., G.W., and S.T.), with discrepancies resolved by a fourth investigator (H.L.). Among the data gleaned details were the surname of the first author, the year of publication, patient age (mean), PD stage, sample size, and intervention details (dose, type, duration). Data were retrieved independently for trials that included more than one experimental arm. When multiple reports referred to the same trial or cohort, the most recent and comprehensive publication was selected for analysis.

#### Bias and quality control

Three independent reviewers (M.Z., Y.Z. and W.Z.) assessed study validity and risk of bias, with disagreements resolved by a fourth reviewer (J.M.). The quality of RCTs was evaluated using the Cochrane RoB 2 tool. Potential publication bias was assessed through visual examination of funnel plots, and Egger’s regression analysis was conducted when the meta-analysis comprised a minimum of 10 studies. If asymmetry was detected, the trim-and-fill procedure was utilized to rectify any publishing bias. A leave-one-out sensitivity analysis was carried out to assess the influence of individual investigations on the overall results.

#### Subgroup and meta-regression analysis

Subgroup and meta-regression analyses were conducted for outcomes with ≥ 10 studies, considering acupuncture-related variables (type, needling sites, number of points, and needle characteristics), session parameters (frequency and total duration), and study location. Both univariable and multivariable meta-regressions were performed using mixed-effects models, with model fit assessed by adjusted R^2^, τ^2^, I^2^ residual, and F-statistics.

#### Certainty assessment

The confidence in proof was assessed employing the Grading of Recommendations, Assessment, Development, and Evaluation (GRADE) approach and categorized into four categories: high, moderate, low, and extremely low [21]. It was conducted only for outcomes with ≥ 3 studies to ensure reliable evidence evaluation.

#### Statistical analysis

Statistical analysis of the data was conducted using RevMan 5.4 (Nordic Cochran Centre, Copenhagen, Denmark) as well as Stata 14 (College Station, TX, USA). This study calculated the pooled standardized mean difference (SMD) for continuous data, accompanied by 95% confidence intervals (CIs), and relative risk (RR) for adverse events. Inter-study heterogeneity was evaluated with I^2^ statistics. A fixed-effects model was used when substantial heterogeneity was absent (I^2^ < 50% or p > 0.05); otherwise, a random-effects model was implemented [22]. Citation handling along with data structuring were conducted via EndNote 21. A p-value of less than 0.05 was considered statistically significant.

## Results

### Study selection

**Fig. 1:**
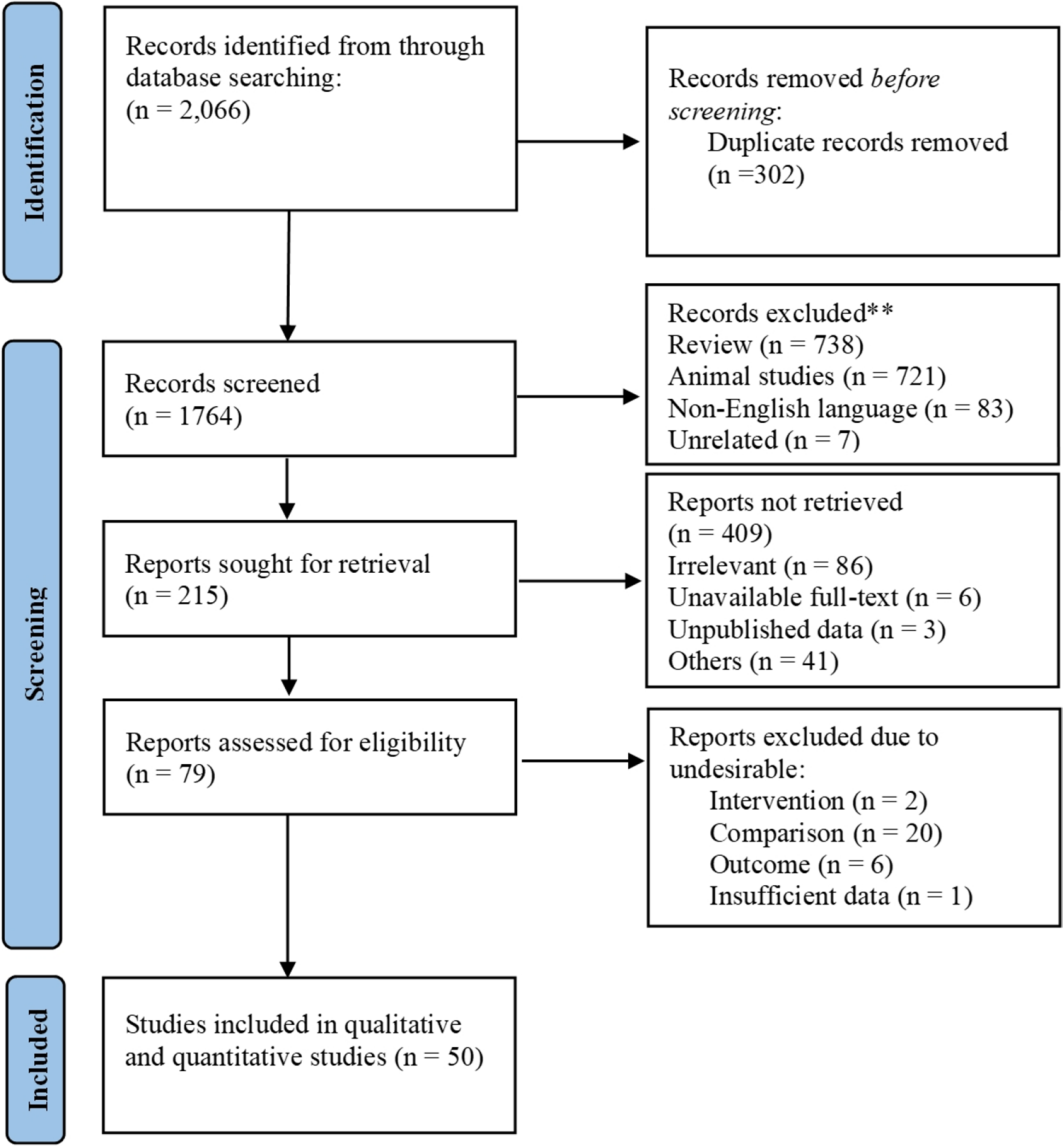
PRISMA flow diagram illustrating the study selection process

Of 2,066 identified records, 302 duplicates were eliminated, leaving 1,764 for screening. After excluding 1,685 records, 79 full-text articles were assessed, with 29 excluded due to intervention (2), comparator (20), outcome (6), or insufficient data (1). Ultimately, 50 studies [11, 23–42] were included (Fig. 1).

### Study characteristics

**Fig. 2:**
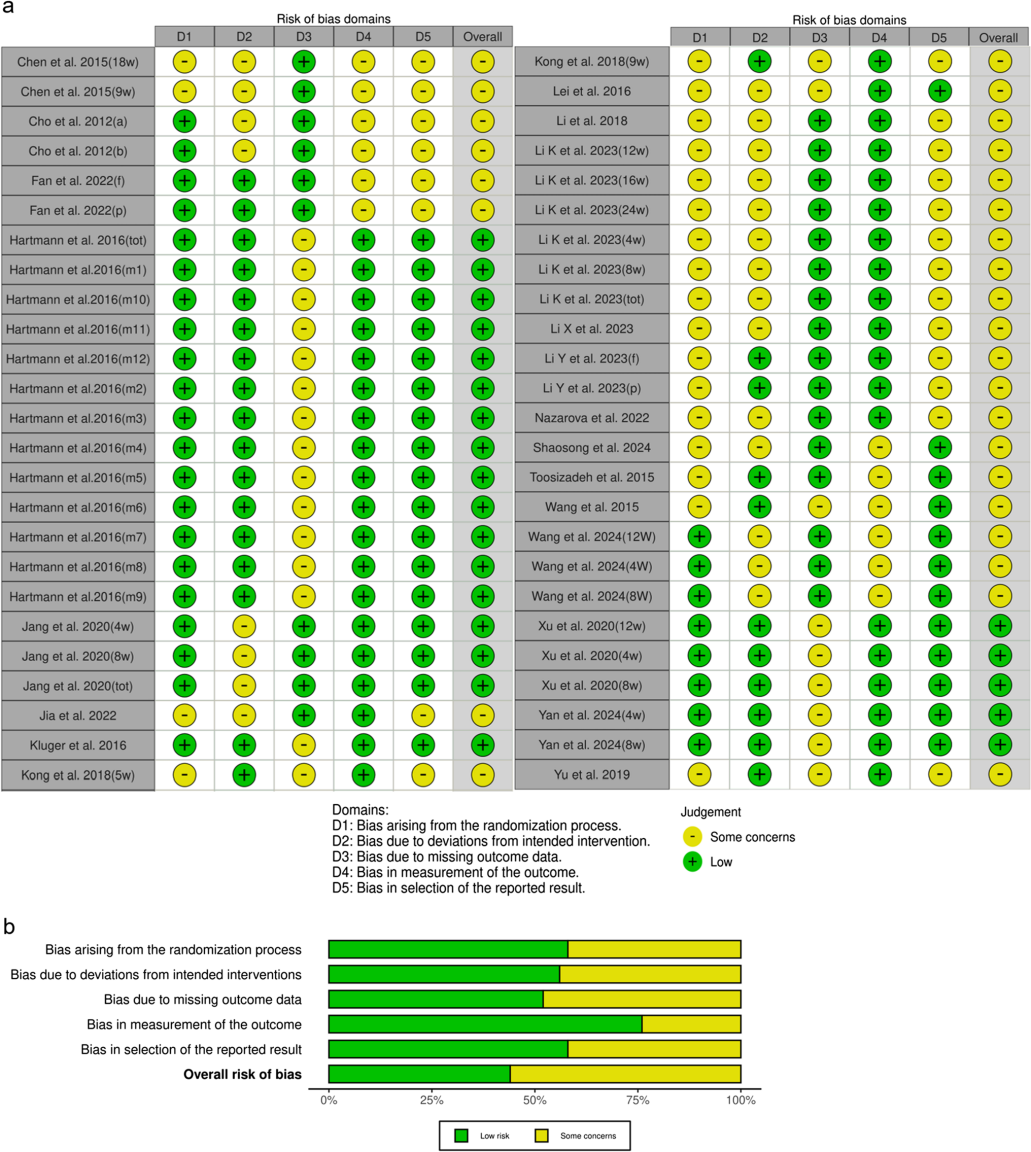
Bias assessment: **a**) summary, **b**) graph

This analysis included 50 RCTs involving patients with PD, aged 40–64.4 years, evaluating various acupuncture modalities—MAC (17), EAC (16), BVAC (14), and FNT (3)—as adjunctive therapies alongside WM. Acupuncture aimed to improve motor and non-motor symptoms, as well as quality of life. Treatment protocols included sessions up to three times weekly for a maximum duration of 16 weeks, using needles with diameters of up to 0.5 mm (Supplementary Table S1). Risk-of-bias assessment indicated that none of the studies had a"high risk,"while 44% demonstrated a"low risk" (Fig. 2).

### UPDRS total (T)

**Fig. 3:**
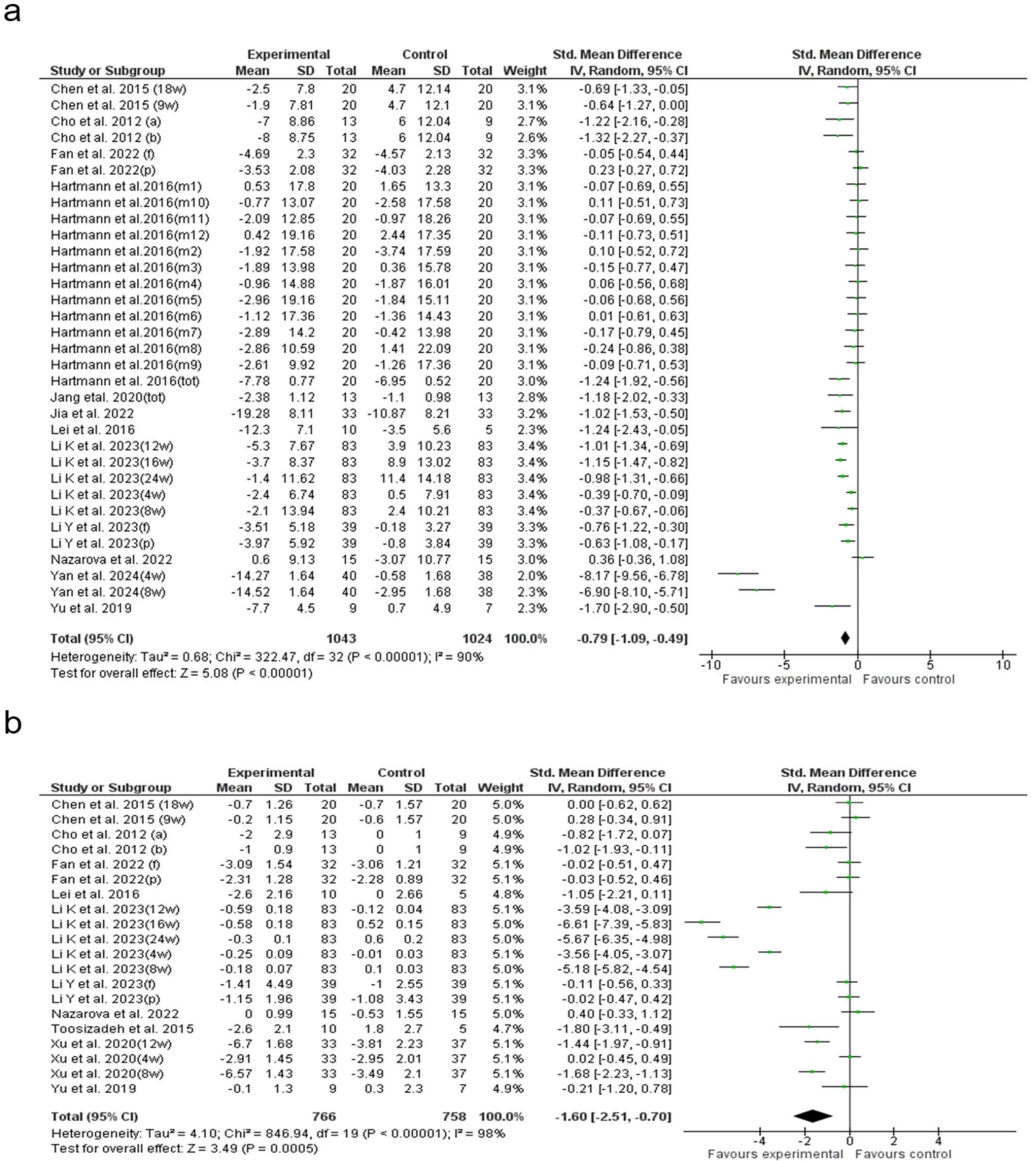
Forest plot of the **a**) UPDRS total and **b**) UPDRS I. UPDRS: Unified Parkinson’s Disease Rating Scale

A meta-analysis of 33 trials showed that acupuncture substantially decreased UPDRS-T scores compared to WM alone (SMD: −0.79; 95% CI: −1.09 to −0.49; p < 0.00001), with high heterogeneity (I^2^ = 90%) (Fig. 3a). Subgroup analysis revealed that ≤ 0.25 mm needles achieved the greatest reduction in UPDRS-T (SMD: −1.66; 95% CI: −2.32 to −1.00), while targeting ≥ 10 acupoints (SMD: −2.18; 95% CI: −2.99 to −1.36) and the abdominal region (SMD: −4.05; 95% CI: −7.34 to −0.75) led to the greatest improvement. Sessions lasting 20–30 min (SMD: −1.36; 95% CI: −1.89 to −0.83) and treatments given three times per week (SMD: −1.56; 95% CI: −2.17 to −0.95) were most effective. MAC had the strongest impact (SMD: −1.76; 95% CI: −2.63 to −0.90), with studies from China reporting the largest reductions (SMD: −1.35) (Supplementary Figs. S1—S2). Univariable meta-regression found needle thickness as the only significant predictor (p = 0.022) (Supplementary Fig. S3a). While, multivariable meta-regression showed that scalp (β = –1.58, p = 0.016), abdominal (β = –1.59, p = 0.023), limb/joint (β = –1.66, p = 0.022), and facial/head stimulation (β = –9.16, p < 0.001) were significantly associated with reductions in UPDRS-T scores. The model explained 93.7% of the between-study variance (p < 0.0001). Egger’s test (p = 0.152) and the funnel plot indicated no publication bias (Supplementary Tables S2-S5 and Fig. S4a).

### UPDRS I

Analysis of 20 studies revealed that acupuncture significantly reduced UPDRS I scores compared to WM alone (SMD = –1.60; 95% CI: –2.51 to –0.70; *p* = 0.0005), despite high heterogeneity (I^2^ = 98%) (Fig. 3b). Subgroup analysis showed that ≤ 0.25 mm needles had the most significant effect (SMD = –2.68), while needling ≥ 10 acupoints further enhanced the outcome (SMD = –4.9). Treating the limb and joint yielded the greatest benefit (SMD = –2.8), with sessions > 20 min (SMD = –1.9) and three sessions per week (SMD = –2.1) proving most effective. EAC showed the strongest impact (SMD = –3), while studies from China reported the highest improvements (SMD = –2.1) (Supplementary Figs. S5—S6). Univariable meta-regression confirmed acupoint number (p = 0.000) and acupuncture type (p = 0.001) as key predictors of better outcomes (Supplementary Fig. S3b-c). Multivariable meta-regression showed that EAC (β = –4.87, p < 0.001), needle sizes ≤ 0.25 mm (β = –3.97, p < 0.001) and ≥ 0.25 mm (β = –4.06, p = 0.003) were significantly associated with greater reductions in UPDRS I scores. In contrast, studies conducted in the United States (U.S.) (β = 4.40, p < 0.001) were associated with attenuated improvements. The model explained 82.98% of the between-study variance (p < 0.0001). Egger’s test (p = 0.976) and the funnel plot indicated no publication bias (Supplementary Tables S5-S8 and Fig. S4b).

### UPDRS II

**Fig. 4:**
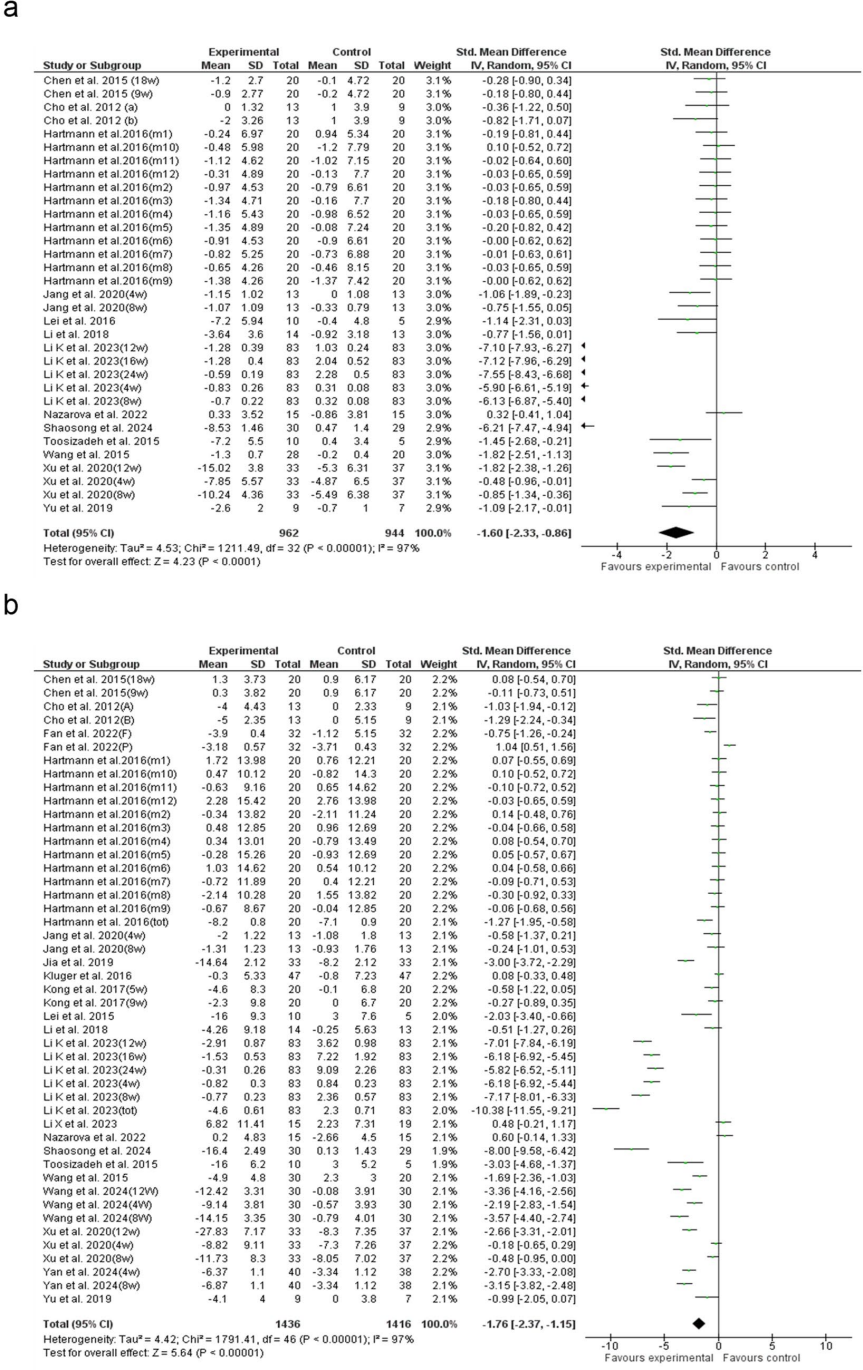
Forest plot of the **a**) UPDRS II and **b**) UPDRS III. UPDRS: Unified Parkinson’s Disease Rating Scale

A pooled analysis of 33 trials showed that acupuncture significantly improved UPDRS II scores compared to WM (SMD: −1.60; 95% CI: −2.33 to −0.86; p < 0.0001), with high heterogeneity (I^2^ = 97%) (Fig. 4a). Subgroup analysis indicated that ≤ 0.25 mm needles had the greatest effect (SMD = –3.66), while ≥ 10 acupoints showed the highest reduction (SMD = –6.73). Limb and joint acupuncture yielded the most improvement (SMD = –4.22), and 20–30-min sessions were the most effective (SMD = –3.27). Three sessions per week maximized benefits (SMD = –4.41), EAC had the highest efficacy (SMD = –3.96), and studies from China (SMD = –3.77) showed the strongest results (Supplementary Figs. S7—S8). Univariable meta-regression confirmed significant associations between treatment effects and needle thickness (β = 0.86, p = 0.024), needling region (β = 0.67, p = 0.006), session frequency (β = −1.06, p = 0.000), acupuncture type (β = −1.36, p = 0.017), and study country (β = 0.82, p = 0.029) (Supplementary Fig. S3d-h). Multivariable meta-regression showed that EAC (β = –2.54, p = 0.005), a stimulation frequency of 3 sessions per week (β = –3.67, p < 0.001), needle sizes ≤ 0.25 mm (β = –5.73, p < 0.001) and 0.25–0.50 mm (β = –2.87, p = 0.008) were significantly associated with improvements in activities of daily living. Studies conducted in the U.S. (β = 3.65, p < 0.001) and session durations of 20–30 min (β = 3.02, p = 0.043) were associated with attenuated benefits. The model explained 83.48% of the between-study variance (p < 0.0001). Egger’s test (p = 0.013) and the funnel plot suggested slight asymmetry (Supplementary Table S5 and Fig. S4c). Trim-and-fill analysis adjusted for missing studies, yielding a corrected SMD of −1.97 (Supplementary Tables S9-S12).

### UPDRS III

The analysis of 43 studies revealed a significant reduction in UPDRS III scores with acupuncture compared to WM (SMD: −1.76; 95% CI: −2.37 to −1.15; p < 0.00001), though heterogeneity was high (I^2^ = 97%) (Fig. 4b). Subgroup analysis revealed that needle thickness significantly influenced outcomes, with ≤ 0.25 mm needles achieving the greatest UPDRS III reduction (SMD = –4.08). The ≥ 10 acupoints subgroup showed the highest effect (SMD = –4.34). Treatment site analysis indicated the most substantial improvement in the back and spine group (SMD = –5.52). Sessions lasting 20–30 min were the most effective (SMD = –3.44), while a frequency of three times per week yielded the greatest reduction (SMD = –4.06). EAC had the highest efficacy among acupuncture types (SMD = –4.09). The most significant treatment effects were observed in studies originating from China (SMD = –3.63), as indicated by country-based analysis (Supplementary Figs. S9—S10). Univariable meta-regression analysis showed significant associations with session frequency per week (β = −1.82, p = 0.000), and study country (β = 0.58, p = 0.019) (Supplementary Table S13 and Fig. S3i—j). Multivariable meta-regression showed that BVAC (β = –397.98, p < 0.001), EAC (β = –250.36, p < 0.001), and FNT (β = –166.51, p < 0.001) were significantly associated with greater reductions in UPDRS III scores. A stimulation frequency of 1–3 sessions per week (β = 112.22, p = 0.002) was associated with attenuated benefits, while 3 sessions per week (β = –43.82, p = 0.009) contributed to greater motor improvement. Among stimulation regions, abdominal (β = 151.70, p < 0.001), limb/joint (β = 357.39, p < 0.001), back/spine (β = 366.72, p < 0.001), and facial/head (β = 247.16, p < 0.001) areas were linked to reduced treatment efficacy. Studies conducted in France (β = 511.58, p < 0.001) were associated with less favorable outcomes, whereas those from Taiwan (β = –152.18, p < 0.001) showed enhanced motor improvement. The model explained 78.03% of the between-study variance (R^2^ = 78.03%, p < 0.0001). The funnel plot indicated minor distortion, and Egger’s test (p < 0.001) pointed to possible publication bias (Supplementary Table S5 and Fig. S4d). After trim-and-fill correction, the adjusted SMD was −2.30 (Supplementary Table S12-S15).

### UPDRS IV

**Fig. 5:**
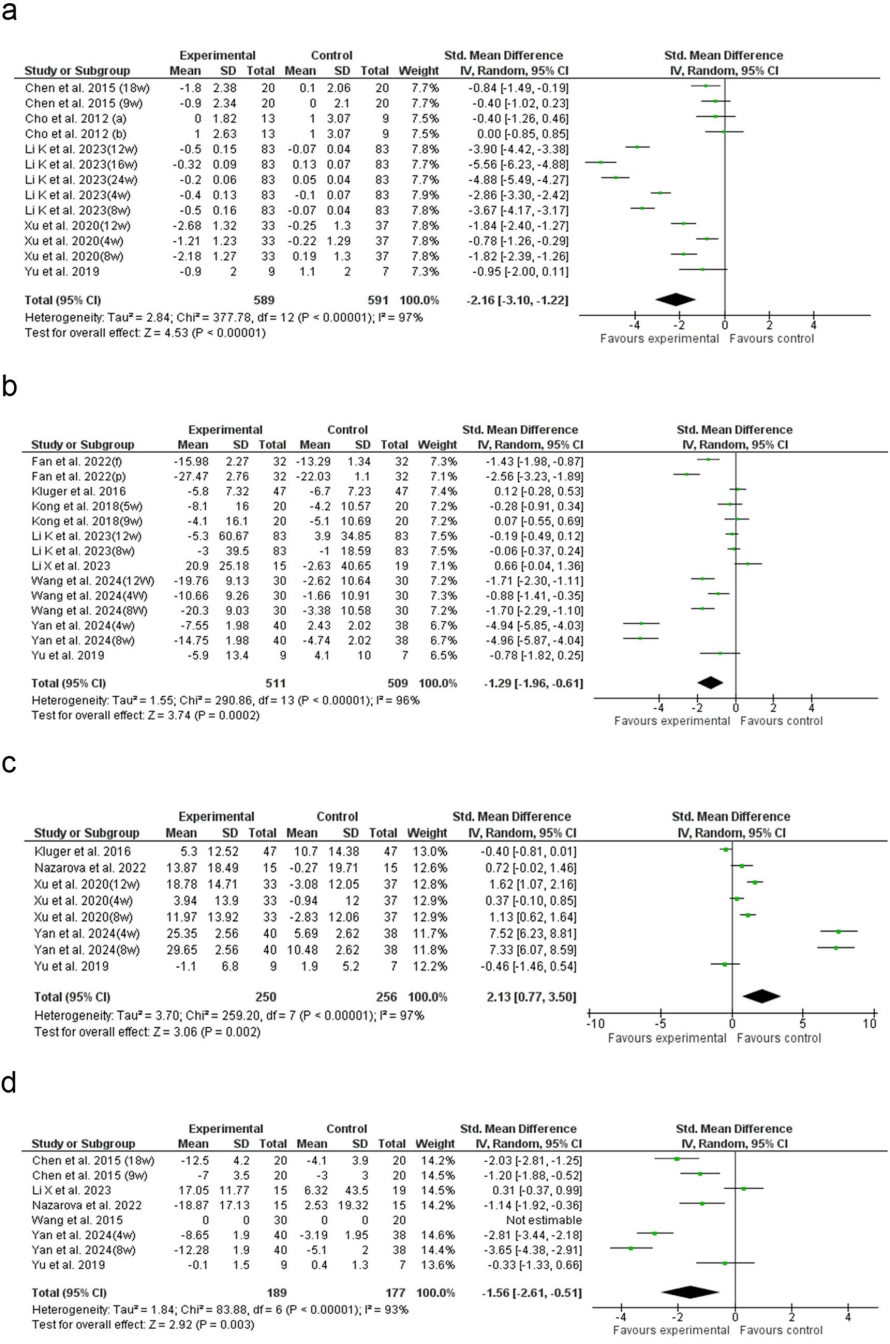
Forest plot of the **a**) UPDRS IV, **b**) PDQ-39, **c**) PDSS and **d**) NMSS. UPDRS: Unified Parkinson’s disease rating scale, PDQ-39: Parkinson’s disease questionnaire-39, PDSS: Parkinson’s disease sleep scale, NMSS: non-motor symptoms scale

The analysis of 13 studies demonstrated a significant reduction in UPDRS IV scores with acupuncture compared to WM (SMD: −2.16; 95% CI: −3.10 to −1.22; p < 0.00001**)**, with high heterogeneity **(**I^2^ = 97%**)** (Fig. 5a). The funnel (Supplementary Fig. S4e) and Egger’s test (p = 0.344) did not indicate significant publication bias (Supplementary Table S5). Subgroup analysis revealed that needle thickness significantly influenced outcomes, with ≤ 0.25 mm needles achieving the greatest UPDRS IV reduction (SMD = –3.06). The ≥ 10 acupoints subgroup showed the highest effect (SMD = –4.15). Treatment site analysis indicated the most substantial improvement in the limb and joint group (SMD = –3.06). Sessions lasting more than 20 min were the most effective (SMD = –2.93), while a frequency of three times per week yielded the greatest reduction (SMD = –2.93). Also, EAC had the highest efficacy among acupuncture types (SMD = –3.15). Country-based analysis highlighted studies conducted in China (SMD = –3.15) and Taiwan (SMD = –0.66) as showing the most significant effects (Supplementary Figs. S11—S12). Univariable meta-regression identified significant effects for acupoints (β = −1.57, p = 0.001), session duration (β = −2.52, p = 0.016), session frequency (β = −2.52, p = 0.016), acupuncture type (β = −1.37, p = 0.018), and study country (β = −1.37, p = 0.018), with EAC and China showing the strongest impact (Supplementary Fig. S13a—e). Multivariable meta-regression showed that session durations > 20 min were significantly associated with greater reductions in UPDRS-IV scores (β = –3.95, p < 0.001), whereas stimulation of the facial and head regions (β = 3.58, p = 0.007) and mixed body regions (β = 3.09, p = 0.003) were associated with less improvement in UPDRS-IV outcomes. The model explained 81.6% of the between-study variance (p < 0.001) (Supplementary Tables S16-S18).

### PDQ-39

The analysis of 14 studies revealed a significant reduction in PDQ-39 scores with acupuncture compared to WM (SMD: −1.29; 95% CI: −1.96 to −0.61; p = 0.0002), though heterogeneity was high (I^2^ = 96%) (Fig. 5b). Subgroup analysis showed that ≤ 0.25 mm needles had the greatest PDQ-39 reduction (SMD = –2.30). Also, the ≥ 10 acupoints subgroup showed the highest effect (SMD = –1.66). Among treatment sites, the abdominal group had the most significant improvement (SMD = –4.95). Sessions lasting 20–30 min were the most effective (SMD = –1.69), while a frequency of three times per week yielded the greatest reduction (SMD = –1.88). Also, MAC had the highest efficacy (SMD = –2.30). Among all countries analyzed, studies from China exhibited the most pronounced effects (SMD = –1.73) (Supplementary Figs. S14—S15). Univariable meta-regression identified significant associations between PDQ-39 improvement and treatment region (β = 1.43, p = 0.002) as well as session frequency (β = −2.03, p = 0.043) (Supplementary Fig. S13f—g). Multivariable meta-regression showed that stimulation of the limb and joint (β = 3.99, p < 0.001), facial and head (β = 3.68, p < 0.001), and mixed body regions (β = 4.17, p = 0.002) were significantly associated with less improvement in PDQ-39 scores. The model explained 82.5% of the between-study variance (p < 0.001). The funnel plot suggested asymmetry (Supplementary Fig. S4f), and Egger’s test (p = 0.012) indicated potential publication bias (Supplementary Table S5). After trim-and-fill correction, the adjusted SMD remained unchanged (SMD: −1.29) (Supplementary Table S12, S19-S21).

### Quality of sleep and non-motor manifestations

The analysis of eight studies showed that acupuncture substantially raised PDSS scores in comparison to WM (SMD: 2.13; 95% CI: 0.77 to 3.50; p = 0.002), although heterogeneity was substantial (I^2^ = 97%). Likewise, eight studies assessing NMSS reported a significant reduction following acupuncture (SMD: −1.56; 95% CI: −2.61 to −0.51; p = 0.003), with high heterogeneity observed (I^2^ = 93%) (Fig. 5c—d).

### Pain and sensory symptoms

The analysis of 11 studies indicated a significant reduction in VAS scores following acupuncture compared to WM (SMD: −1.71; 95% CI: −2.50 to −0.93; p < 0.0001), with considerable heterogeneity observed (I^2^ = 97%) (Supplementary Fig. S16a). Inequalities in the funnel plot and the results of Egger’s test (p = 0.003) suggested the possibility of publication bias (Supplementary Table S5 and Fig. S4h). Trim-and-fill analysis suggested no missing studies; the effect estimate (SMD = −1.714) remained unchanged (Supplementary Table S12).

### Mood and psychological well-being

The aggregated analysis of five trials evaluating BD-II scores revealed no substantial distinction between acupuncture and WM (SMD: −0.21; 95% CI: −0.54 to 0.13; p = 0.22; I^2^ = 0%). In contrast, five studies evaluating HAM-D reported a significant reduction following acupuncture (SMD: −0.87; 95% CI: −1.59 to −0.16; p = 0.02), though heterogeneity remained high (I^2^ = 87%). Similarly, six studies measuring HAM-A demonstrated a significant improvement (SMD: −2.30; 95% CI: −4.06 to −0.53; p = 0.01), despite considerable heterogeneity (I^2^ = 98%) (Supplementary Fig. S16b—d).

### Disease severity

The analysis of 2 studies found no significant difference in Hoehn and Yahr stages between acupuncture and WM (SMD: 0.00; 95% CI: −0.72 to 0.72; p = 1.00) (Supplementary Fig. S16e).

### Gait and balance function

The meta-analysis of three studies found no significant effect of acupuncture on cadence (SMD: −0.58; 95% CI: −1.49 to 0.34; p = 0.22), with substantial heterogeneity (I^2^ = 80%). Similarly, stride length showed no significant improvement in two studies (SMD: 0.15; 95% CI: −0.39 to 0.70; p = 0.59), with no observed heterogeneity (I^2^ = 0%). However, stride time, based on two studies, significantly improved following acupuncture (SMD: 0.83; 95% CI: 0.26 to 1.40; p = 0.004), with no heterogeneity detected (I^2^ = 0%). Additionally, velocity (SMD: −0.37; 95% CI: −0.92 to 0.18; p = 0.18) and gait freezing (SMD: −0.53; 95% CI: −1.08 to 0.03; p = 0.06) showed no significant improvement, with no heterogeneity (I^2^ = 0%). Lastly, BBS scores, evaluated in two studies, demonstrated no significant change (SMD: −0.88; 95% CI: −2.66 to 0.90; p = 0.33), with high heterogeneity (I^2^ = 86%) (Supplementary Fig. S17a—f).

### Adverse event

Among the 24 eligible studies, 13 (54.2%) reported no adverse events in either group. Meta-analysis of the remaining trials showed a non-significant risk of adverse events in the acupuncture group (RR = 1.51; 95% CI: 0.93–2.46; P = 0.10; I^2^ = 23%). Most reported events were mild and self-limiting, such as subcutaneous hematoma, bruising, needling pain, dizziness, nausea, and itching. A few studies noted more distressing but reversible symptoms, including sharp or prolonged pain, allergic reactions, and treatment-related discomforts. No serious adverse events were reported (Supplementary Table S22 and Fig. S18).

### Sensitivity analysis

The sensitivity evaluation validated the reliability of the results, since the omission of any individual research did not substantially affect the total meta-analysis values (Supplementary Fig. S19).

### Grading of evidence

According to the GRADE assessment, the quality of evidence varied across outcomes. UPDRS-T, UPDRS I, UPDRS IV, PDSS, NMSS, HAM-A, HAM-D, and Cadence were rated as moderate. UPDRS II, UPDRS III, VAS and PDQ-39 were classified as low. Significantly, BD-II was the only result classified as high-quality (Supplementary Table S23).

## Discussion

This systematic review and meta-analysis offer compelling evidence for the effectiveness of acupuncture in PD rehabilitation, showing marked improvements in both motor and non-motor symptoms. The consistent decline in UPDRS-T scores reflects a broad neuromodulatory effect, further clarified by subgroup analyses highlighting needle diameter ≤ 0.25 mm, use of > 10 acupoints, and abdominal stimulation as key clinical enhancers. Also, MAC stood out for its precise afferent targeting and mechanosensory activation [43]. These results align with preclinical studies showing acupuncture modulates dopaminergic signaling, reduces neuroinflammation, and boosts neurotrophic factor expression [44]. Thinner needles may be more effective due to selective stimulation of low-threshold Aβ fibers, avoiding Aδ and C nociceptive activation, which enhances parasympathetic tone and reduces discomfort—thus optimizing dorsal column–medial lemniscal system engagement. Neurophysiological evidence shows such input enhances brain plasticity in areas relevant to PD, like the basal ganglia and supplementary motor area [45, 46]. Likewise, using > 10 acupoints across various regions may boost therapeutic response via spatial summation and greater central integration of multisite input, promoting thalamocortical modulation and improved motor-limbic connectivity. Multiregional stimulation could also activate separate autonomic and somatosensory reflex arcs, supporting broader systemic effects [47]. Regional efficacy—especially abdominal and limb/joint stimulation—may reflect differences in neuroanatomical pathways: abdominal input influences vagal and enteric-central circuits, potentially addressing neuroimmune and gastrointestinal-motor disruptions in PD, while limb/joint input targets somatomotor pathways, aiding rigidity and bradykinesia [48]. This was further supported by multivariable meta-regression, showing that stimulation of the scalp, abdomen, limbs/joints, and especially the facial/head region significantly contributed to UPDRS-T improvement. These areas likely activate distinct neurofunctional pathways, with facial/head stimulation offering the strongest effect due to its direct access to cranial-autonomic circuits and central integrative hubs, enabling broad neuromodulatory impact. Notably, the most robust improvements were consistently reported in studies conducted in China, likely reflecting greater procedural uniformity, routine implementation of EAC, and integration of standardized clinical protocols that closely adhere to stimulation parameters optimized for both physiological engagement and therapeutic benefit [49, 50].

Therapeutic gains were also prominent in the non-motor domain, with UPDRS I scores showing notable reductions in symptoms like cognitive decline, depression, apathy, and autonomic issues. EAC demonstrated the strongest results, likely due to its steady neuromodulatory input across peripheral and central circuits [51]. Evidence suggests EAC boosts parasympathetic activity via vagal afferent activation and moderates hypothalamic output, reducing hypothalamic–pituitary–adrenal axis overactivity and lowering cortisol levels [52]. This may enhance stress resilience and stabilize mood, positively influencing UPDRS I elements tied to motivation and emotional health [16, 53]. Limb and joint acupoint stimulation also improved autonomic function, likely through somatosensory afferents reaching autonomic hubs like the nucleus tractus solitarius and parabrachial nucleus, which regulate cardiovascular, digestive, and temperature control—functions often impaired in PD [54]. Acupuncture’s modulation of monoaminergic systems, especially serotonin and dopamine, further supports its neurochemical benefits in mood and cognition. Animal research indicates it raises serotonin in the hippocampus and prefrontal cortex, potentially easing depression and cognitive decline in PD models [55]. These effects align with observed increases in functional plasticity in motor and non-motor cortical regions after acupuncture [56]. Multivariable analysis also showed that EAC and both thin and moderately thick needles were strongly associated with UPDRS I improvement, suggesting that stimulation modality and afferent dynamics shape non-motor outcomes. EAC likely enhances limbic-autonomic regulation via rhythmic, low-frequency input, while needle size variation may recruit complementary fiber types (Aβ, Aδ), broadening neuromodulatory effects [56]. As seen with motor outcomes, thinner needles and targeting more than ten acupoints yielded superior effects—likely due to enhanced Aβ fiber recruitment and cumulative afferent integration within limbic-autonomic circuits. Furthermore, longer sessions and thrice-weekly treatments may allow for sustained neuromodulatory entrainment, reinforcing central patterning in emotional and autonomic pathways essential for mitigating symptoms such as dysphoria. Notably, studies from China reported the most consistent and effective outcomes. In contrast, studies from the U.S. showed reduced efficacy—likely reflecting differences in clinical practice. U.S. acupuncture is often individualized, with lower use of EAC, less treatment frequency, and considerable heterogeneity in technique and training [57]. These factors may reduce afferent signal consistency and engagement of central circuits, emphasizing the need for procedural standardization to optimize non-motor symptom relief in PD.

Acupuncture also improved functional independence, reflected in UPDRS II score reductions. Subgroup analysis showed that thinner needles, wider acupoint coverage, and limb/joint-focused stimulation yielded greater clinical benefit. Mechanistically, thin needles likely activate Aβ fibers without pain input, enhancing spinal and cortical plasticity key to motor control [58, 59]. Broader stimulation may enable spatial summation, improving sensorimotor integration across neural circuits [60]. Targeting limbs and joints—rich in proprioceptors—might further reinforce corticospinal links and neuromuscular control, directly influencing UPDRS II functions like walking, turning, and dressing [14]. Multivariable analysis revealed that EAC, moderate needle sizes, thrice-weekly sessions, and 20–30-min durations significantly contributed to better functional outcomes in daily activities. These parameters likely support consistent afferent signaling, engaging both somatomotor and regulatory networks involved in task initiation and physical autonomy—mechanisms overlapping with those implicated in non-motor improvements. The strong effect of thin to moderate needles may reflect optimized sensory recruitment, while a balanced session schedule facilitates adaptive plasticity without inducing central fatigue. Despite the high efficacy observed in Chinese studies, reduced efficacy in U.S.-based studies may stem from inconsistent use of these parameters, limiting the intensity and integration of therapeutic input.

EAC showed superior efficacy in enhancing motor performance, reflected by significant UPDRS III score reductions. This benefit stems from EAC’s ability to modulate cortical excitability and boost dopaminergic signaling via peripheral nerve stimulation and improving motor circuit function [61]. It helps restore balance in the basal ganglia-thalamocortical loop and suppress excessive GABAergic output, thereby enhancing coordination [61]. Also, stimulating acupoints along the spine, such as DU-14 and BL-23, likely engages central pattern generators and spinal interneurons, aiding postural control and gait [62]. This strategy enhances proprioceptive input and neuromuscular integration, key elements in motor rehabilitation. Similarly, the superior motor outcomes associated with thinner needles, higher acupoint counts, and structured session timing may reflect improved sensorimotor mapping, temporal synchrony in motor planning, and enhanced treatment regularity—factors critical for restoring execution dynamics in Parkinsonian movement. Consistently, studies from China, where clinical delivery tends to be more protocol-driven, yielded the strongest effects. Accordingly, univariable meta-regression linked better outcomes to higher session frequency and certain geographic settings. Additional modeling indicated BVAC, EAC, and FNT as the strongest predictors of motor improvement (UPDRS III). BVAC may exert its effects through melittin and apamin, known to inhibit microglial activation and oxidative stress, while EAC and FNT likely enhance neuroplasticity via repeated, concentrated sensory activation [10]. The superior outcomes observed with thrice-weekly sessions suggest a dose–response dynamic critical for strengthening motor feedback loops. Notably, studies from Taiwan reported significantly greater motor improvements, likely reflecting structured clinical routines, frequent use of EAC and fire needling, and practitioner training grounded in embodied skills and tactile sensitivity [63]. In contrast, weaker effects in French studies may stem from less frequent treatments, minimal use of electrostimulation, and a predominantly theoretical training model focused on short postgraduate courses with limited hands-on emphasis [64]. These regional differences illustrate how practice structure, therapeutic intensity, and skill transmission shape the efficacy of acupuncture in motor rehabilitation.

Beyond primary motor gains, acupuncture notably reduced levodopa-induced dyskinesia and motor fluctuations, as shown by UPDRS IV scores. EAC again proved most effective, likely due to its modulation of striatal glutamate signaling, suppression of hyperkinetic oscillations, and stabilization of dopaminergic firing within cortico-basal ganglia pathways [61, 65]. This balancing of excitatory–inhibitory dynamics may be key to mitigating abnormal motor outputs from chronic levodopa use. Stimulation of limb and joint acupoints showed the most consistent benefits. While also effective in UPDRS II and III contexts, here its role likely involves enhancing proprioceptive input, recalibrating motor cortical excitability, and improving thalamic relay synchronization [66]. This somatosensory feedback may help suppress involuntary movement through descending inhibition and spinal reflex control. Consistent with improvements reported in UPDRS I–III and total motor burden, the greatest reductions in UPDRS IV occurred with ≤ 0.25 mm needles, ≥ 10 acupoints, and limb/joint targeting. These configurations may fine-tune sensorimotor loops and suppress dyskinetic activity by stabilizing aberrant basal ganglia output. Longer, thrice-weekly sessions likely support dopaminergic equilibrium and reduce motor fluctuations. As with earlier outcomes, Chinese trials yielded the most consistent effects, reflecting structured and intensive intervention delivery. Meta-regression added insight into procedural factors: longer sessions (> 20 min) and more acupoints correlated with better outcomes, possibly due to prolonged activation of endogenous opioids and endocannabinoids, both of which modulate motor pathways [67]. In contrast, facial or head acupoint targeting was less effective, perhaps due to trigeminal hypersensitivity or overstimulation of brainstem areas. Overall, findings suggest acupuncture modulates dyskinesia through both anatomical targeting and systemic neuromodulatory effects that stabilize cortico-striatal circuitry. These results support the use of tailored, duration-adjusted, and context-aware acupuncture strategies for managing advanced motor symptoms in PD [17].

In addition to easing motor symptoms, acupuncture enhanced overall quality of life in PD, as reflected in PDQ-39 outcomes. MAC achieved the greatest PDQ-39 benefit, likely by aligning sensory input with dynamic non-motor symptom profiles through tailored stimulation. Also, the marked effect of abdominal stimulation underscores the relevance of the gut–brain axis in PD pathophysiology [30, 68]. Prior studies showed acupuncture alters gut microbiota and vagal tone, contributing to anti-inflammatory effects and better patient-reported outcomes [8, 69]. The most pronounced PDQ-39 improvements were observed in subgroups receiving thinner needles, ≥ 10 acupoints, and regular 20–30 min sessions administered thrice weekly. These parameters may enhance sensory salience and procedural regularity, thereby fostering more consistent engagement of affective, autonomic, and interoceptive circuits central to perceived quality of life in PD. The amplified outcomes seen in Chinese studies further underscore the relevance of treatment standardization and cultural alignment in optimizing patient-centered benefits. Nevertheless, multivariable meta-regression showed reduced efficacy with limb/joint, facial/head, and mixed-region stimulation. These areas may insufficiently engage networks mediating interoception, emotion, or autonomic tone—domains central to PDQ-39. Limb inputs, for example, primarily activate somatic-motor fibers with limited relevance to mood or fatigue; facial/head stimulation, while triggering cranial circuits, may bypass the gut–limbic pathways essential for quality-of-life modulation. Mixed targeting also could dilute afferent specificity, weakening central integration. Recent neuroimaging and clinical evidence indicates that abdominal and auricular stimulation more reliably engages the nucleus tractus solitarius, locus coeruleus, and insular cortex—key centers for affective-autonomic integration in PD [54]. Complementary findings further validate the clinical relevance of anatomically targeted EAC in enhancing PDQ-39 outcomes [70, 71]. These observations collectively underscore the importance of circuit-specific input in optimizing non-motor therapeutic effects.

Parallel benefits were observed in sleep-related outcomes, as evidenced by significant gains in PDSS scores. EAC was the most effective modality, likely due to its ability to regulate melatonin secretion and balance autonomic nervous system activity [72]. These findings align with prior studies suggesting that acupuncture enhances slow-wave sleep and reduces sleep fragmentation by modulating the suprachiasmatic nucleus [19]. Given the high prevalence of sleep disturbances in PD, acupuncture may serve as an effective non-pharmacological intervention for improving sleep architecture. Additionally, the significant reductions in NMSS scores suggest a broad impact on non-motor symptoms, including autonomic dysfunction and mood disorders.

Consistent with its broader neuromodulatory effects, acupuncture also reduced pain perception in PD, as evidenced by improvements in VAS scores. Mechanistic studies suggest that these analgesic effects may be mediated through endogenous opioid release, suppression of pro-inflammatory cytokines, and alterations in spinal pain processing networks [13]. Given the increasing recognition of pain as a debilitating symptom in PD, acupuncture may provide an alternative or adjunct to pharmacological pain management strategies [73]. The reduction in HAM-D and HAM-A scores suggests that acupuncture exerts anxiolytic and antidepressant effects, although variability in treatment response was observed. MAC demonstrated greater efficacy for anxiety symptoms, while EAC appeared to be more effective for depression. These differences may be attributed to variations in cortical excitability modulation and limbic system regulation [74]. Prior studies have indicated that acupuncture increases hippocampal serotonin and brain-derived neurotrophic factor levels, which may contribute to its antidepressant effects [16]. However, the absence of significant effects on BD-II scores suggests that acupuncture’s impact on bipolar disorder symptoms may be limited.

The lack of significant differences in Hoehn and Yahr staging indicates that while acupuncture improves symptom severity, it may not alter disease progression. Similarly, the absence of substantial improvements in cadence, stride length, and balance function suggests that acupuncture has a limited impact on gait mechanics in PD. However, the significant enhancement in stride time suggests that acupuncture may improve locomotor rhythm, possibly through its effects on cerebellar and basal ganglia circuits [53, 75]. Also, the lack of adverse event reports in the majority of included studies highlights acupuncture’s consistently favorable safety profile in PD management. When present, adverse effects were sporadic, mild, and transient—most commonly minor bruising, dizziness, or localized discomfort—indicating superficial physiological responses rather than systemic complications. The uniformity of these outcomes across diverse intervention protocols suggests negligible modality-specific risk. However, this observed safety consistency underscores the need for rigorous and standardized adverse event reporting in future trials to strengthen comparative safety assessments.

Overall, these findings suggest that EAC protocols using more than 10 acupoints, with targeted stimulation of abdominal and limb/joint regions and needle size matched to symptom domain (thinner for non-motor, moderate for motor), yield the most consistent benefits across motor outcomes, functional independence, dyskinesia, and quality of life in PD. Regimens involving three weekly sessions lasting 20–30 min were most effective in these domains. Acupuncture as practiced in China closely reflects this therapeutic profile, distinguished by several clinical advantages: integration within hospital-based care, routine use of EAC with precise frequency and waveform settings, individualized point selection grounded in classical diagnostics, and dual training of practitioners in both TCM and WM. These factors likely contribute to stronger afferent signal reliability, deeper neuromodulatory engagement, and greater procedural consistency—mechanisms that align directly with the intervention features identified as most effective in our analyses. In clinical practice, adopting these optimized parameters—particularly those modeled on Chinese procedural standards—may enhance the efficacy, reproducibility, and personalization of acupuncture-based interventions for PD.

Despite the promising findings, several limitations should be acknowledged. A key limitation lies in the substantial heterogeneity observed across studies, which likely stems from variations in acupuncture techniques, patient characteristics, treatment protocols, and outcome measures. Moreover, methodological challenges inherent to the included RCTs may have influenced the overall reliability of the findings. Blinding procedures remain particularly problematic in acupuncture research due to the sensory nature of the intervention, increasing the risk of performance and detection bias. While some trials employed single-blinding strategies, full double-blinding was rarely feasible. In addition, allocation concealment was often inadequately reported, raising concerns about potential selection bias. Although most studies utilized standardized outcome measures such as the UPDRS and PDQ-39, inconsistency in assessment timing, evaluator blinding, and follow-up duration may have contributed to further variability in outcomes. While sensitivity analyses confirmed the robustness of the results, the presence of publication bias in certain domains suggests the need for cautious interpretation. The GRADE assessment also revealed that the overall certainty of evidence ranged from moderate to low across most outcomes. These findings underscore the importance of conducting future high-quality, multicenter RCTs with rigorous methodological designs, adequate blinding and allocation procedures, and harmonized outcome assessments. Also, incorporating patient-reported outcomes and preferences alongside clinical indicators is also essential to foster a more comprehensive and patient-centered evaluation of acupuncture’s therapeutic role in PD.

## Conclusion

This meta-analysis underscores the therapeutic precision of acupuncture as an adjunctive strategy in PD. EAC demonstrated robust efficacy for non-motor symptoms and treatment-related motor complications, while MAC—particularly with abdominal targeting—consistently improved quality of life. Optimal outcomes were associated with thinner needles, broader acupoint engagement, and high-frequency protocols, most notably in standardized Chinese trials. These findings support anatomically guided, symptom-specific acupuncture as a clinically relevant intervention and highlight the imperative for rigorously designed, culturally attuned studies to advance its integration into PD care.

## Supplementary Information

Below is the link to the electronic supplementary material.

Supplementary file1 (DOCX 82 KB)

Supplementary file2 (DOCX 4856 KB)

Supplementary file3 (DOCX 33 KB)

## Data Availability

Data sharing not applicable to this article as no datasets were generated or analyzed during the current study.
