# Supplementary material for: Efficacy of various acupuncture modalities on alleviating symptoms in Parkinson’s disease: a systematic review and meta-analysis of randomized controlled trials
Source: Neurol Sci. 2025 Jul 17;46(10):4819–35. doi: 10.1007/s10072-025-08333-1 (PMC12488745; doi:10.1007/s10072-025-08333-1)
Supplement: Supplementary file 1 — Supplementary file1 (DOCX 82 KB) [file 10072_2025_8333_MOESM1_ESM.docx]

**Table S1.** Characteristics of the included studies.

| Study | Country | Sex (M/F) | Population | Mean Age | Intervention_ Exp | Intervention_ Ctrl | Acupuncture Points | Treatment Duration (W) | Needle Thickness | Primary Location | Frequency (Sessions/W) | Total Sessions |
| --- | --- | --- | --- | --- | --- | --- | --- | --- | --- | --- | --- | --- |
| Chen et al. 2015(18w), 18W follow up | Taiwan | 40/40 | 80 | 45 | MAC + WM | WM | 8 | 12 | 0.27 × 25 mm, 0.27 × 40 mm | Abdominal | 3 | 36 |
| Chen et al. 2015(9w), 9W follow up | Taiwan | 40/40 | 80 | 45 | MAC + WM | WM | 8 | 12 | 0.27 × 25 mm, 0.27 × 40 mm | Abdominal | 3 | 36 |
| Cho et al. 2012(a) | South Korea | 30/30 | 60 | 50 | MAC + WM | WM | 5 | 8 | 0.25 × 30 mm | Limb & Joint | 2 | 16 |
| Cho et al. 2012(b) | South Korea | 30/30 | 60 | 50 | BVAC + WM | WM | 5 | 8 | 0.25 × 30 mm | Limb & Joint | 2 | 16 |
| Fan et al. 2022(f), final assessment | China | 25/25 | 50 | 48 | MAC + WM | WM | 5 | 6 | 0.25 × 25 mm, 0.25 × 40 mm | Limb & Joint | 2 | 12 |
| Fan et al. 2022(p), post treatment | China | 25/25 | 50 | 48 | MAC + WM | WM | 5 | 6 | 0.25 × 25 mm, 0.25 × 40 mm | Limb & Joint | 2 | 12 |
| Hartmann et al. 2016 (tot), total | France | 20/20 | 40 | 55 | BVAC + WM | WM | 1 | 1 | 25G Hypodermic Needle ≈ 0.50 mm | Mixed Body | < 1 | 11 |
| Hartmann et al.2016(m1), 1M follow up | France | 20/20 | 40 | 55 | BVAC + WM | WM | 1 | 1 | 25G Hypodermic Needle ≈ 0.50 mm | Mixed Body | < 1 | 11 |
| Hartmann et al.2016(m10), 10M follow up | France | 20/20 | 40 | 55 | BVAC + WM | WM | 1 | 1 | 25G Hypodermic Needle ≈ 0.50 mm | Mixed Body | < 1 | 11 |
| Hartmann et al.2016(m11), 11M follow up | France | 20/20 | 40 | 55 | BVAC + WM | WM | 1 | 1 | 25G Hypodermic Needle ≈ 0.50 mm | Mixed Body | < 1 | 11 |
| Hartmann et al.2016(m12), 12M follow up | France | 20/20 | 40 | 55 | BVAC + WM | WM | 1 | 1 | 25G Hypodermic Needle ≈ 0.50 mm | Mixed Body | < 1 | 11 |
| Hartmann et al.2016(m2), 2M follow up | France | 20/20 | 40 | 55 | BVAC + WM | WM | 1 | 1 | 25G Hypodermic Needle ≈ 0.50 mm | Mixed Body | < 1 | 11 |
| Hartmann et al.2016(m3), 3M follow up | France | 20/20 | 40 | 55 | BVAC + WM | WM | 1 | 1 | 25G Hypodermic Needle ≈ 0.50 mm | Mixed Body | < 1 | 11 |
| Hartmann et al.2016(m4), 4M follow up | France | 20/20 | 40 | 55 | BVAC + WM | WM | 1 | 1 | 25G Hypodermic Needle ≈ 0.50 mm | Mixed Body | < 1 | 11 |
| Hartmann et al.2016(m5), 5M follow up | France | 20/20 | 40 | 55 | BVAC + WM | WM | 1 | 1 | 25G Hypodermic Needle ≈ 0.50 mm | Mixed Body | < 1 | 11 |
| Hartmann et al.2016(m6), 6M follow up | France | 20/20 | 40 | 55 | BVAC + WM | WM | 1 | 1 | 25G Hypodermic Needle ≈ 0.50 mm | Mixed Body | < 1 | 11 |
| Hartmann et al.2016(m7), 7M follow up | France | 20/20 | 40 | 55 | BVAC+ WM | WM | 1 | 1 | 25G Hypodermic Needle ≈ 0.50 mm | Mixed Body | < 1 | 11 |
| Hartmann et al.2016(m8), 8M follow up | France | 20/20 | 40 | 55 | BVAC + WM | WM | 1 | 1 | 25G Hypodermic Needle ≈ 0.50 mm | Mixed Body | < 1 | 11 |
| Hartmann et al.2016(m9), 9M follow up | France | 20/20 | 40 | 55 | BVAC + WM | WM | 1 | 1 | 25G Hypodermic Needle ≈ 0.50 mm | Mixed Body | < 1 | 11 |
| Jang et al. 2020(4w), 4W follow up | South Korea | 35/35 | 70 | 52 | MAC + WM | WM | NS | NS | NS | Mixed Body | NS | NS |
| Jang et al. 2020(8w), 8W follow up | South Korea | 35/35 | 70 | 52 | MAC + WM | WM | NS | NS | NS | Mixed Body | NS | NS |
| Jang et al. 2020(tot), total | South Korea | 35/35 | 70 | 52 | MAC + WM | WM | NS | NS | NS | Mixed Body | NS | NS |
| Jia et al. 2022 | China | 50/50 | 100 | 42 | MAC + WM | WM | 13 | 16 | 0.30 × 40 mm | Scalp | 3 | 48 |
| Kluger et al. 2016 | USA | 59/35 | 94 | 64.4 | MAC + WM | WM | 11 | 6 | 0.2 x 25.4 | Mixed Body | 2 | 12 |
| Kong et al. 2018(5w), 5W follow up | Singapore | 45/45 | 90 | 40 | EAC + WM | WM | 11 | 10 | 70 mm Park Sham Needle & Real Needle | Facial & Head | 3 | 30 |
| Kong et al. 2018(9w), 9W follow up | Singapore | 45/45 | 90 | 40 | EAC+ WM | WM | 11 | 10 | 71 mm Park Sham Needle & Real Needle | Facial & Head | 3 | 30 |
| Lei et al. 2016 | USA | 30/30 | 60 | 47 | EAC + WM | WM | 9 | 8 | 0.25 × 40-50 mm | Limb & Joint | 2 | 16 |
| Li K et al. 2023(12w), 12W follow up | China | 40/40 | 80 | 49 | EAC + WM | WM | 10 | 12 | 0.25 × 25 mm | Limb & Joint | 3 | 36 |
| Li K et al. 2023(16w), 16W follow up | China | 40/40 | 80 | 49 | EAC + WM | WM | 10 | 12 | 0.25 × 25 mm | Limb & Joint | 3 | 36 |
| Li K et al. 2023(24w), 24W follow up | China | 40/40 | 80 | 49 | EAC + WM | WM | 10 | 12 | 0.25 × 25 mm | Limb & Joint | 3 | 36 |
| Li K et al. 2023(4w), 4W follow up | China | 40/40 | 80 | 49 | EAC + WM | WM | 10 | 12 | 0.25 × 25 mm | Limb & Joint | 3 | 36 |
| Li K et al. 2023(8w), 8W follow up | China | 40/40 | 80 | 49 | EAC + WM | WM | 10 | 12 | 0.25 × 25 mm | Limb & Joint | 3 | 36 |
| Li K et al. 2023(tot), total | China | 40/40 | 80 | 49 | EAC + WM | WM | 10 | 12 | 0.25 × 25 mm | Limb & Joint | 3 | 36 |
| Li X et al. 2023 | China | 25/25 | 50 | 53 | MAC + WM | WM | NS | NS | 0.50 × 50 mm | Unspecified | NS | NS |
| Li Y et al. 2023(f), final assessment | China | 42/43 | 85 | 46 | MAC + WM | WM | 5 | 10 | 0.30 × 25 mm, 0.30 × 40 mm | Scalp | 2 | 20 |
| Li Y et al. 2023(p), post treatment | China | 42/43 | 85 | 46 | MAC + WM | WM | 5 | 10 | 0.30 × 25 mm, 0.30 × 40 mm | Scalp | 2 | 20 |
| Li et al. 2018 | China | 48/47 | 95 | 51 | MAC + WM | WM | 3 | 6 | 0.25 × 20-30 mm | Scalp | 2 | 12 |
| Nazarova et al. 2022 | China | 60/60 | 120 | 50 | EAC + WM | WM | 8 | 12 | 0.30 × 75 mm | Scalp-Abdominal | 3 | 36 |
| Shaosong et al. 2024 | China | 45/45 | 90 | 44 | EAC + WM | WM | 6 | 8 | 0.30 × 40 mm | Back & Spine | 2 | 16 |
| Toosizadeh et al. 2015 | USA | 50/50 | 100 | 49 | EAC + WM | WM | 9 | 10 | 0.25 × 40-50 mm | Back & Spine | 2 | 20 |
| Wang et al. 2015 | China | 37/38 | 75 | 47 | EAC + WM | WM | 4 | 6 | 0.25 × 40 mm | Limb & Joint | 2 | 12 |
| Wang et al. 2024(12W), 12W follow up | China | 32/28 | 60 | 62.17 | FNT + WM | WM | 5 | 5 | 0.40mm x 40mm | Facial & Head | 3 | 8 |
| Wang et al. 2024(4W), 4W follow up | China | 32/28 | 60 | 62.17 | FNT + WM | WM | 5 | 5 | 0.40mm x 40mm | Facial & Head | 3 | 8 |
| Wang et al. 2024(8W), 8W follow up | China | 32/28 | 60 | 62.17 | FNT + WM | WM | 5 | 5 | 0.40mm x 40mm | Facial & Head | 3 | 8 |
| Xu et al. 2020(12w), 12W follow up | China | 42/43 | 85 | 46 | EAC + WM | WM | 4 | 6 | NS | Mixed Body | 2 | 12 |
| Xu et al. 2020(4w), 4W follow up | China | 42/44 | 85 | 46 | EAC + WM | WM | 4 | 6 | NS | Mixed Body | 2 | 12 |
| Xu et al. 2020(8w), 8W follow up | China | 42/45 | 85 | 46 | EAC + WM | WM | 4 | 6 | NS | Mixed Body | 2 | 12 |
| Yan et al. 2024(4w), 4W follow up | China | 55/55 | 110 | 48 | MAC+ WM | WM | 10 | 12 | 0.25 × 25 mm, 0.25 × 40 mm | Abdominal | 3 | 36 |
| Yan et al. 2024(8w), 8W follow up | China | 55/55 | 110 | 48 | MAC + WM | WM | 10 | 12 | 0.25 × 25 mm, 0.25 × 40 mm | Abdominal | 3 | 36 |
| Yu et al. 2019 | Taiwan | 30/35 | 65 | 50 | MAC + WM | WM | 3 | 8 | 0.30 × 40 mm | Facial & Head | 2 | 16 |

M/F: male/female, W: week, M: month, MA: manual acupuncture, WM: western medicine, BVAC: bee venom acupuncture, EAC: electroacupuncture, FNT: fire needle therapy, NS: not specified

**Table S2.** Univariable meta-regression analysis of UPDRS total across key variables.

| Variable | Coefficient | Standard error | p-value | t-value | 95% CI | Tau^2^ |
| --- | --- | --- | --- | --- | --- | --- |
| Needle specification | 0.7084 | 0.2945 | 0.022* | 2.41 | [0.1078, 1.3089] | 2.134 |
| Acupuncture areas | 0.3831 | 0.1974 | 0.061 | 1.94 | [-0.0194, 0.7857] | 2.301 |
| Session duration | 0.5426 | 0.4069 | 0.192 | 1.33 | [-0.2873, 1.3725] | 2.459 |
| Session frequency | -0.4277 | 0.2665 | 0.119 | -1.6 | [-0.9728, 0.1174] | 1.279 |
| Acupuncture type | 0.6564 | 0.3884 | 0.101 | 1.69 | [-0.1358, 1.4487] | 2.392 |
| Needling frequency | 0.1382 | 0.2571 | 0.595 | 0.54 | [-0.3861, 0.6626] | 2.608 |
| Country | 0.1475 | 0.2891 | 0.614 | 0.51 | [-0.4422, 0.7372] | 2.618 |

UPDRS: Unified Parkinson’s Disease Rating Scale, CI: confidence interval, *p-value < 0.05

**Table S3.** Summary of multivariable meta-regression coefficients for predictors of UPDRS total.

| Category | Predictors | Coefficient | Standard error | t-value | p-value > \|t \| | 95% CI |
| --- | --- | --- | --- | --- | --- | --- |
| Acupuncture type | MAC | 1.053892 | 0.6117948 | 1.72 | 0.085 | [-0.1452034, 2.252988] |
|  | EAC | 0.6177739 | 0.5858828 | 1.05 | 0.292 | [-0.5305353, 1.766083] |
| Acupuncture areas | Scalp | -1.577812 | 0.6579245 | -2.40 | 0.016* | [-2.867321, -0.2883039] |
|  | Abdominal | -1.588457 | 0.6983158 | -2.27 | 0.023* | [-2.957131, -0.2197835] |
|  | Limb & Joint | -1.663889 | 0.7239276 | -2.30 | 0.022* | [-3.082761, -0.2450174] |
|  | Facial & Head | -9.160656 | 0.9838361 | -9.31 | 0.000* | [-11.08894, -7.232373] |
| Needle specification | NR | -2.127999 | 0.814078 | -2.61 | 0.009* | [-3.723562, -0.5324352] |
|  | ≤ 0.25 mm | 0.4328036 | 0.432482 | 1.00 | 0.317 | [-0.4148457, 1.280453] |
|  | _cons | -0.1422413 | 0.1218035 | -1.17 | 0.243 | [-0.3809717, 0.0964892] |

**Table S4.** Model fit statistics for multivariable meta-regression of UPDRS total.

| Number of studies | Tau² | I² residual (%) | Adjusted R² (%) | Wald chi² (8) | p-value |
| --- | --- | --- | --- | --- | --- |
| 31 | 0.0913 | 57.98 | 93.7 | 123.47 | 0.0000* |

**Tabe S5.** Egger's test results for assessing publication bias.

| Outcome | No. of studies | Egger's Intercept | p-value |
| --- | --- | --- | --- |
| UPDRS-T | 33 | -2.18 | 0.152 |
| UPDRS I | 20 | -4.63 | 0.408 |
| UPDRS II | 33 | -12.046 | 0.013* |
| UPDRS III | 45 | -13.384 | < 0.001* |
| UPDRS IV | 13 | 6.963 | 0.344 |
| PDQ-39 | 14 | -8.205 | 0.012* |
| VAS | 11 | -8.453 | 0.003* |

PDQ-39: Parkinson’s Disease Questionnaire-39, VAS: Visual Analogue Scale

**Table S6.** Univariable meta-regression analysis of UPDRS I across key variables.

| Variable | Coefficient | Standard error | p-value | t-value | 95% CI | Tau^2^ |
| --- | --- | --- | --- | --- | --- | --- |
| Needle specification | 0.9571 | 0.7207 | 0.201 | 1.33 | [-0.5571, 2.4712] | 4.346 |
| Needling frequency | -1.9818 | 0.3675 | 0* | -5.39 | [-2.7539, -1.2098] | 1.749 |
| Acupuncture areas | -0.1313 | 0.3268 | 0.693 | -0.4 | [-0.8180, 0.5553] | 4.735 |
| Session duration | -1.5235 | 1.1881 | 0.216 | -1.28 | [-4.0197, 0.9727] | 4.367 |
| Session frequency | -0.6496 | 0.7421 | 0.393 | -0.88 | [-2.2086, 0.9094] | 4.567 |
| Acupuncture type | -1.5593 | 0.3733 | 0.001* | -4.18 | [-2.3435, -0.7751] | 2.356 |
| Country | 0.556 | 0.4572 | 0.237 | 1.22 | [-0.4010, 1.5202] | 4.394 |

**Table S7.** Summary of multivariable meta-regression coefficients for predictors of UPDRS I.

| Category | Predictors | Coefficient | Standard Error | t-value | p-value > \|t\| | 95% CI |
| --- | --- | --- | --- | --- | --- | --- |
| Acupuncture type | BVAC | -0.9348313 | 0.9924093 | -0.94 | 0.346 | [–2.879918, 1.010255] |
|  | EAC | -4.873395 | 0.8035562 | -6.06 | 0.000* | [–6.448336, –3.298454] |
| Needle specification | ≤ 0.25 mm | -3.974221 | 0.7323168 | -5.43 | 0.000* | [–5.409535, –2.538906] |
|  | ≥ 0.25 mm | -4.062074 | 1.375663 | -2.95 | 0.003* | [–6.758324, –1.365824] |
| Country | Tiawan | 0.2153888 | 1.100892 | 0.20 | 0.845 | [–1.94232, 2.373097] |
|  | USA | 4.402141 | 0.8382173 | 5.25 | 0.000* | [2.759265, 6.045016] |
|  | _cons | 3.949783 | 0.9960198 | 3.97 | 0.000* | [1.99762, 5.901945] |

**Table S8.** Model fit statistics for multivariable meta-regression of UPDRS I.

| Number of studies | Tau² | I² residual (%) | Adjusted R² (%) | Wald chi² (6) | p-value |
| --- | --- | --- | --- | --- | --- |
| 18 | 0.849 | 89.10 | 82.98 | 78.83 | 0.0000* |

**Table S9.** Univariable meta-regression analysis of UPDRS II across key variables.

| Variable | Coefficient | Standard error | p-value | t-value | 95% CI | Tau^2^ |
| --- | --- | --- | --- | --- | --- | --- |
| Needle specification | 0.8631 | 0.3639 | 0.024* | 2.37 | [0.1209, 1.6052] | 5.208 |
| Needling frequency | 0.4074 | 0.3491 | 0.252 | 1.17 | [-0.3047, 1.1195] | 5.932 |
| Acupuncture areas | 0.668 | 0.2272 | 0.006* | 2.94 | [0.2047, 1.1313] | 4.799 |
| Session duration | 0.851 | 0.5492 | 0.131 | 1.55 | [-0.2689, 1.9710] | 5.742 |
| Session frequency | -1.0572 | 0.2712 | 0* | -3.9 | [-1.6103, -0.5039] | 4.105 |
| Acupuncture type | -1.362 | 0.5399 | 0.017* | -2.52 | [-2.4631, -0.2609] | 5.121 |
| Country | 0.8171 | 0.3565 | 0.029* | 2.29 | [0.0901, 1.5441] | 5.286 |

**Table S10.** Summary of multivariable meta-regression coefficients for predictors of UPDRS II.

| Category | Predictors | Coefficient | Standard error | t-value | p-value > \|t\| | 95% CI |
| --- | --- | --- | --- | --- | --- | --- |
| Acupuncture type | BVAC | 2.483214 | 1.415582 | 1.75 | 0.079 | [-0.2912762, 5.257704] |
|  | EAC | -2.543973 | 0.9082722 | -2.80 | 0.005 | [-4.324153, -0.7637918] |
| Session frequency | 1<<3 x/w | 0.4979584 | 1.357344 | 0.37 | 0.714 | [-2.162387, 3.158304] |
|  | 3x/w | -3.670917 | 0.9764803 | -3.76 | 0.000 | [-5.584784, -1.757051] |
| Needle specification | ≤ 0.25 mm | -5.73039 | 0.7710946 | -7.43 | 0.000 | [-7.241708, -4.219073] |
|  | 0.25<<0.50 mm | -2.869763 | 1.087662 | -2.64 | 0.008 | [-5.001542, -0.737984] |
|  | ≥0.50 mm | -2.290516 | 1.497367 | -1.53 | 0.126 | [-5.225032, 0.6442694] |
| Session duration | 20<≤30 min | 3.017972 | 1.489994 | 2.03 | 0.043 | [0.0976367, 5.938807] |
|  | NR | -2.196084 | 1.608527 | -1.37 | 0.172 | [-5.34874, 0.9565714] |
| Country | France | -2.26656 | 1.342748 | -1.69 | 0.091 | [-4.898298, 0.3651773] |
|  | USA | 3.651529 | 0.9454819 | 3.86 | 0.000 | [1.798419, 5.50464] |
|  | _cons | 2.138334 | 1.885641 | 1.13 | 0.257 | [-1.557454, 5.834122] |

**Table S11.** Model fit statistics for multivariable meta-regression of UPDRS II.

| Number of studies | Tau² | I² residual (%) | Adjusted R² (%) | Wald chi² (11) | p-value |
| --- | --- | --- | --- | --- | --- |
| 31 | 1.003 | 89.64 | 83.48 | 147.87 | 0.0000* |

**Table S12.** Trim and Fill analysis summary.

| Outcome | Model | Observed effect size (95% CI) | Imputed studies | Adjusted effect size (95% CI) |
| --- | --- | --- | --- | --- |
| UPDRS II | Random-effects | -1.595 (-2.334 to -0.857) | 6 | -1.970 (-2.673 to -1.267) |
| UPDRS III | Random-effects | -1.846 (-2.481 to -1.211) | 8 | -2.301 (-2.946 to -1.656) |
| PDQ-39 | Random-effects | -1.289 (-1.965 to -0.613) | 0 | -1.289 (-1.965 to -0.613) |
| VAS | Random-effects | -1.714 (-2.496 to -0.932) | 0 | -1.714 (-2.496 to -0.932) |

**Table S13.** Univariable meta-regression analysis of UPDRS III across key variables.

| Variable | Coefficient | Standard error | p-value | t-value | 95% CI | Tau^2^ |
| --- | --- | --- | --- | --- | --- | --- |
| Needle specification | 0.637 | 0.3667 | 0.089 | 1.74 | [-1.1019, 1.3758] | 6.369 |
| Needling frequency | 0.4521 | 0.3319 | 0.18 | 1.36 | [-0.2172, 1.1214] | 6.567 |
| Acupuncture areas | -0.5277 | 0.3412 | 0.129 | -1.55 | [-1.2159, 0.1605] | 6.503 |
| Session duration | 0.1526 | 0.5102 | 0.766 | 0.3 | [-0.8762, 1.1815] | 6.843 |
| Session frequency | -1.8238 | 0.3717 | 0* | -4.91 | [-2.5734, -1.0741] | 4.265 |
| Acupuncture type | -0.8401 | 0.4176 | 0.051 | -2.01 | [-1.6823, 0.0021] | 6.236 |
| Country | 0.5827 | 0.2396 | 0.019* | 2.43 | [0.0995, 1.0658] | 5.975 |

**Table S14.** Summary of multivariable meta-regression coefficients for predictors of UPDRS III response.

| Category | Predictors | Coefficient | Standard error | t-value | p-value > \|t\| | 95% CI |
| --- | --- | --- | --- | --- | --- | --- |
| Acupuncture type | BVAC | -397.9766 | 95.61641 | -4.16 | 0.000* | [-585.3813, -210.5719] |
|  | EAC | -250.357 | 69.97828 | -3.58 | 0.000* | [-387.5119, -113.2021] |
|  | FNT | -166.5071 | 45.80897 | -3.63 | 0.000* | [-256.291, -76.72319] |
| Needling frequency | 5<<10 | -34.8326 | 18.52685 | -1.88 | 0.060 | [-71.14457, 1.479365] |
|  | ≥10 | -56.61727 | 9.659862 | -5.86 | 0.000* | [-75.55025, -37.68428] |
|  | NR | -394.6639 | 95.61485 | -4.13 | 0.000* | [-582.0655, -207.2622] |
| Session frequency | 1<<3 x/w | 112.2197 | 36.90078 | 3.04 | 0.002* | [39.89546, 184.5439] |
|  | 3 x/w | -43.82103 | 16.71621 | -2.62 | 0.009* | [-76.5842, -11.05787] |
| Acupuncture areas | Abdominal | 151.7017 | 36.14629 | 4.20 | 0.000* | [80.85626, 222.5471] |
|  | Limb & Joint | 357.3947 | 101.4271 | 3.52 | 0.000* | [158.0238, 556.7656] |
|  | Back & Spine | 342.7196 | 72.40821 | 4.73 | 0.000* | [200.5338, 533.3748] |
|  | Facial & Head | 269.7468 | 74.37186 | 3.63 | 0.000* | [120.0113, 418.2098] |
|  | Mixed Body | 161.8782 | 22.00824 | 7.36 | 0.000* | [118.2473, 205.5092] |
|  | Other | -32.40703 | 22.51161 | -1.44 | 0.153 | [-73.82241, 11.00834] |
| Country | France | 511.5848 | 130.3306 | 3.93 | 0.000* | [256.1415, 767.0281] |
|  | Tiawan | -152.1782 | 42.37427 | -3.59 | 0.000* | [-235.2302, -69.12612] |
| _cons | _cons | -77.02914 | 45.57288 | -1.69 | 0.091 | [-166.3503, 12.29206] |

**Table S15.** Model fit statistics for multivariable meta-regression of UPDRS III.

| Number of studies | Tau² | I² residual (%) | Adjusted R² (%) | Wald chi² (16) | p-value |
| --- | --- | --- | --- | --- | --- |
| 29 | 226.3 | 99.99 | 78.03 | 171.67 | 0.0000* |

**Table S16.** Univariable meta-regression analysis of UPDRS IV across key variables.

| Variable | Coefficient | Standard error | p-value | t-value | 95% CI | Tau^2^ |
| --- | --- | --- | --- | --- | --- | --- |
| Needle specification | 0.364 | 0.7799 | 0.65 | 0.47 | [-1.3526, 2.0805] | 3.527 |
| Needling frequency | -1.5652 | 0.3411 | 0.001* | -4.59 | [-2.3160, -0.8140] | 1.161 |
| Acupuncture areas | 0.1669 | 0.5291 | 0.758 | 0.32 | [-0.9977, 1.3315] | 3.563 |
| Session duration | -2.5159 | 0.8812 | 0.016* | -2.86 | [-4.4550, -0.5768] | 2.008 |
| Session frequency | -2.5159 | 0.8812 | 0.016* | -2.86 | [-4.4550, -0.5768] | 2.008 |
| Acupuncture type | -1.3661 | 0.4935 | 0.018* | -2.77 | [-2.4524, -0.2798] | 2.074 |
| Country | 1.3661 | 0.4935 | 0.018* | 2.77 | [0.2798, 2.4524] | 2.074 |

**Table S17.** Summary of multivariable meta-regression coefficients for predictors of UPDRS IV.

| Category | Predictors | Coefficient | Standard error | t-value | p-value > \|t\| | 95% CI |
| --- | --- | --- | --- | --- | --- | --- |
| Session duration | > 20 min | -3.951281 | 0.7319595 | -5.40 | 0.000* | [-5.385895, -2.516667] |
| Acupuncture areas | Limb & Joint | 0.4202521 | 0.8688517 | 0.48 | 0.629 | [-1.282666, 2.12317] |
|  | Facial & Head | 3.579397 | 1.335831 | 2.68 | 0.007* | [0.9612161, 6.197579] |
|  | Mixed Body | 3.092049 | 1.058047 | 2.92 | 0.003* | [1.018316, 5.165783] |
| _cons | _cons | -0.6281165 | 0.5969431 | -1.05 | 0.293 | [-1.798104, 0.5418706] |

**Table S18.** Model fit statistics for multivariable meta-regression of UPDRS IV.

| Number of studies | Tau² | I² residual (%) | Adjusted R² (%) | Wald chi² (4) | p-value |
| --- | --- | --- | --- | --- | --- |
| 13 | 0.6071 | 87.87 | 81.59 | 48.71 | 0.0000* |

**Table S19.** Univariable meta-regression analysis of PDQ-39 across key variables.

| Variable | Coefficient | Standard error | p-value | t-value | 95% CI | Tau^2^ |
| --- | --- | --- | --- | --- | --- | --- |
| Needle specification | 0.3376 | 0.5921 | 0.579 | 0.57 | [-0.9524, 1.6276] | 3.066 |
| Needling frequency | 0.0371 | 0.5067 | 0.943 | 0.07 | [-1.0666, 1.1408] | 3.146 |
| Acupuncture areas | 1.4262 | 0.3498 | 0.002* | 4.08 | [0.6641, 2.1883] | 1.259 |
| Session duration | -0.1552 | 1.0635 | 0.886 | -0.15 | [-2.4723, 2.1619] | 3.144 |
| Session frequency | -2.025 | 0.8972 | 0.043* | -2.26 | [-3.9799, -0.0702] | 2.151 |
| Acupuncture type | 0.5528 | 0.6087 | 0.382 | 0.91 | [-0.7736, 1.8791] | 2.947 |
| Country | 0.6749 | 0.4989 | 0.201 | 1.35 | [-0.4120, 1.7620] | 2.709 |

**Table S20.** Summary of multivariable meta-regression coefficients for predictors of PDQ-39.

| Category | Predictors | Coefficient | Standard error | t-value | p-value > \|t\| | 95% CI |
| --- | --- | --- | --- | --- | --- | --- |
| Session frequency | 3 x/w | -1.20988 | 0.684872 | -1.77 | 0.077 | [-2.552205, 0.1324441] |
| Acupuncture areas | Limb & Joint | 3.994708 | 0.7127977 | 5.60 | 0.000* | [2.59765, 5.391766] |
|  | Facial & Head | 3.681601 | 0.7242339 | 5.08 | 0.000* | [2.262128, 5.101073] |
|  | Other | 4.169999 | 1.064257 | 3.92 | 0.000* | [2.084093, 6.255905] |
| _cons | _cons | -3.78868 | 0.9128827 | -4.15 | 0.000* | [-5.577898, -1.999463] |

**Table S21.** Model fit statistics for multivariable meta-regression of PDQ-39.

| Number of studies | Tau² | I² residual (%) | Adjusted R² (%) | Wald chi² (4) | p-value |
| --- | --- | --- | --- | --- | --- |
| 14 | 0.5171 | 87.65 | 82.53 | 52.14 | 0.0000* |

**Table S22.** Summary of reported adverse events across study groups in the included trials.

| Study | Acupuncture modality + WM (N) | WM (N) |
| --- | --- | --- |
| Cho et al. 2012(a) | No AEs | No AEs |
| Cho et al. 2012(b) | Itching (1) | No AEs |
| Fan et al. 2022(p) | Hematoma (2)  Muscle spasm and difficulty removing the needle (2) | No AEs |
| Hartmann et al. 2016(tot) | Positive skin test, injection site redness/itching, nausea, fatigue, insomnia, dyskinesia | Nausea, fatigue, insomnia, dyskinesia, bradycardia |
| Jang et al. 2020(4w) | No AEs | No AEs |
| Jang et al. 2020(8w) | No AEs | No AEs |
| Jia et al. 2022 | Mild nausea (3), occasional insomnia (1) | Mild nausea (4), excessive appetite (2) |
| Kluger et al. 2016 | Constipation (1) | No AEs |
| Kong et al. 2018(5w) | No AEs | No AEs |
| Kong et al. 2018(9w) | No AEs | No AEs |
| Lei et al. 2016 | Transient dizziness (1) | No AEs |
| Li et al. 2018 | No AEs | No AEs |
| Li K et al. 2023(tot) | Local bruising (6), abdominal pain (2), dizziness (1), local hematoma (1), shingles (1) | Headache (2), dizziness (1), fever (2), diarrhea (1) |
| Li X et al. 2023 | No AEs | No AEs |
| Li Y et al. 2023(p) | Bleeding (3), subcutaneous hematoma (2), sharp pain (1) | No AEs |
| Nazarova et al. 2022 | No AEs | No AEs |
| Shaosong et al. 2024 | Mild bleeding and subcutaneous hematoma | No AEs |
| Toosizadeh et al. 2015 | No AEs | No AEs |
| Wang et al. 2015 | No AEs | No AEs |
| Wang et al. 2024(12W) | Nausea (1), lower limb swelling (1), palpitations (1), subcutaneous hematoma (related to dry needle) (3) | Nausea (4), lower limb swelling (1), orthostatic hypotension (2), palpitations (2) |
| Xu et al. 2020(12w) | No AEs | No AEs |
| Yan et al. 2024(4w) | Nausea, severe sharp pain, prolonged pain over 10 minutes, hematoma, bleeding/numbness/infection, severe tremor during treatment, allergy, other discomforts after acupuncture | Severe pain and tremor, allergy, other discomforts |
| Yan et al. 2024(8w) | No AEs | No AEs |
| Yu et al. 2019 | No AEs | No AEs |

AEs: adverse events

**Table S23.** GRADE assessment of certainty and importance of outcomes.

| Outcome | No of studies | Risk of Bias  (RoB 2 tool) | Inconsistency (I^2^) | Indirectness | Imprecision | Publication Bias  (Eggers) | No of patients | | Certainty | Importance |
| --- | --- | --- | --- | --- | --- | --- | --- | --- | --- | --- |
|  |  |  |  |  |  |  | Acp+WM | WM |  |  |
| UPDRS-T | 33 | Not serious | Serious ^a^ | Not serious | Not serious | Not serious | 1043 | 1024 | ⊕⊕⊕◯ (Moderate) | Critical |
| UPDRS I | 20 | Not serious | Serious ^a^ | Not serious | Not serious | Not serious | 766 | 758 | ⊕⊕⊕◯ (Moderate) | Critical |
| UPDRS II | 33 | Not serious | Serious ^a^ | Not serious | Not serious | Serious ^b^ | 962 | 944 | ⊕⊕◯◯ (Low) | Critical |
| UPDRS III | 45 | Not serious | Serious ^a^ | Not serious | Not serious | Serious ^b^ | 1372 | 1352 | ⊕⊕◯◯ (Low) | Critical |
| UPDRS IV | 13 | Not serious | Serious ^a^ | Not serious | Not serious | Not serious | 589 | 591 | ⊕⊕⊕◯ (Moderate) | Critical |
| PDQ-39 | 14 | Not serious | Serious ^a^ | Not serious | Not serious | Serious ^b^ | 511 | 509 | ⊕⊕◯◯ (Low) | Critical |
| PDSS | 7 | Not serious | Serious ^a^ | Not serious | Not serious | Undetected ^c^ | 250 | 256 | ⊕⊕⊕◯ (Moderate) | Important |
| NMSS | 8 | Not serious | Serious ^a^ | Not serious | Not serious | Undetected ^c^ | 189 | 177 | ⊕⊕⊕◯ (Moderate) | Critical |
| BD-II | 5 | Not serious | Not serious | Not serious | Not serious | Undetected ^c^ | 75 | 65 | ⊕⊕⊕⊕ (High) | Important |
| HAM-A | 6 | Not serious | Serious ^a^ | Not serious | Not serious | Undetected ^c^ | 221 | 207 | ⊕⊕⊕◯ (Moderate) | Important |
| HAM-D | 5 | Not serious | Serious ^a^ | Not serious | Not serious | Undetected ^c^ | 147 | 136 | ⊕⊕⊕◯ (Moderate) | Important |
| Cadence | 3 | Not serious | Serious ^a^ | Not serious | Not serious | Undetected ^c^ | 59 | 59 | ⊕⊕⊕◯ (Moderate) | Critical |
| VAS | 11 | Not serious | Serious ^a^ | Not serious | Not serious | Serious ^b^ | 555 | 544 | ⊕⊕◯◯ (Low) | Important |

^a^ Serious inconsistency due to high heterogeneity (I² > 50%).

^b^ Serious concern of publication bias due to asymmetry in the funnel plot and significant Egger’s test results.

^c^ Fewer than 10 studies, so publication bias could not be reliably assessed.

Acp: acupuncture, PDSS: Parkinson’s disease sleep scale, NMSS: non-motor symptoms scale, BD-II: bipolar disorder-II, HAM: Hamilton anxiety rating scale
