# Supplementary material for: Efficacy of various acupuncture modalities on alleviating symptoms in Parkinson’s disease: a systematic review and meta-analysis of randomized controlled trials
Source: Neurol Sci. 2025 Jul 17;46(10):4819–35. doi: 10.1007/s10072-025-08333-1 (PMC12488745; doi:10.1007/s10072-025-08333-1)
Supplement: Supplementary file 2 — Supplementary file2 (DOCX 4856 KB) [file 10072_2025_8333_MOESM2_ESM.docx]

**Fig. S1** Subgroup analysis of UPDR total based on a) needle specification, b) needling frequency, c) acupuncture areas and (d) session duration. UPDRS: Unified Parkinson’s Disease Rating Scale.

**Fig. S2.** Subgroup analysis of UPDRS total based on a) session frequency, (b) acupuncture type and (c) country.

**Fig. S3** Meta-regression analysis showing the association between UPDRS total with (a) needle specification, the UPDRS I with (b) needling frequency and (c) acupuncture type, the UPDRS II with (d) needle specification, (e) acupuncture areas, (f) session frequency, (g) acupuncture type and (h) country, the UPDRS III with (i) session frequency and (j) country. Each bubble represents a study, with bubble size reflecting the precision of the estimate (inverse of within-study variance). The blue line indicates the regression trend. PDQ-39: Parkinson’s Disease Questionnaire-39.

**Fig. S4** Funnel plot of a) UPDRS total, b) UPDRS I, c) UPDRS II, d) UPDRS III, e) UPDRS IV, f) PDQ-39 and g) VAS. VAS: visual analog scale.

**Fig. S5** Subgroup analysis of UPDRS I based on a) needle specification, b) needling frequency, c) acupuncture areas and (d) session duration.


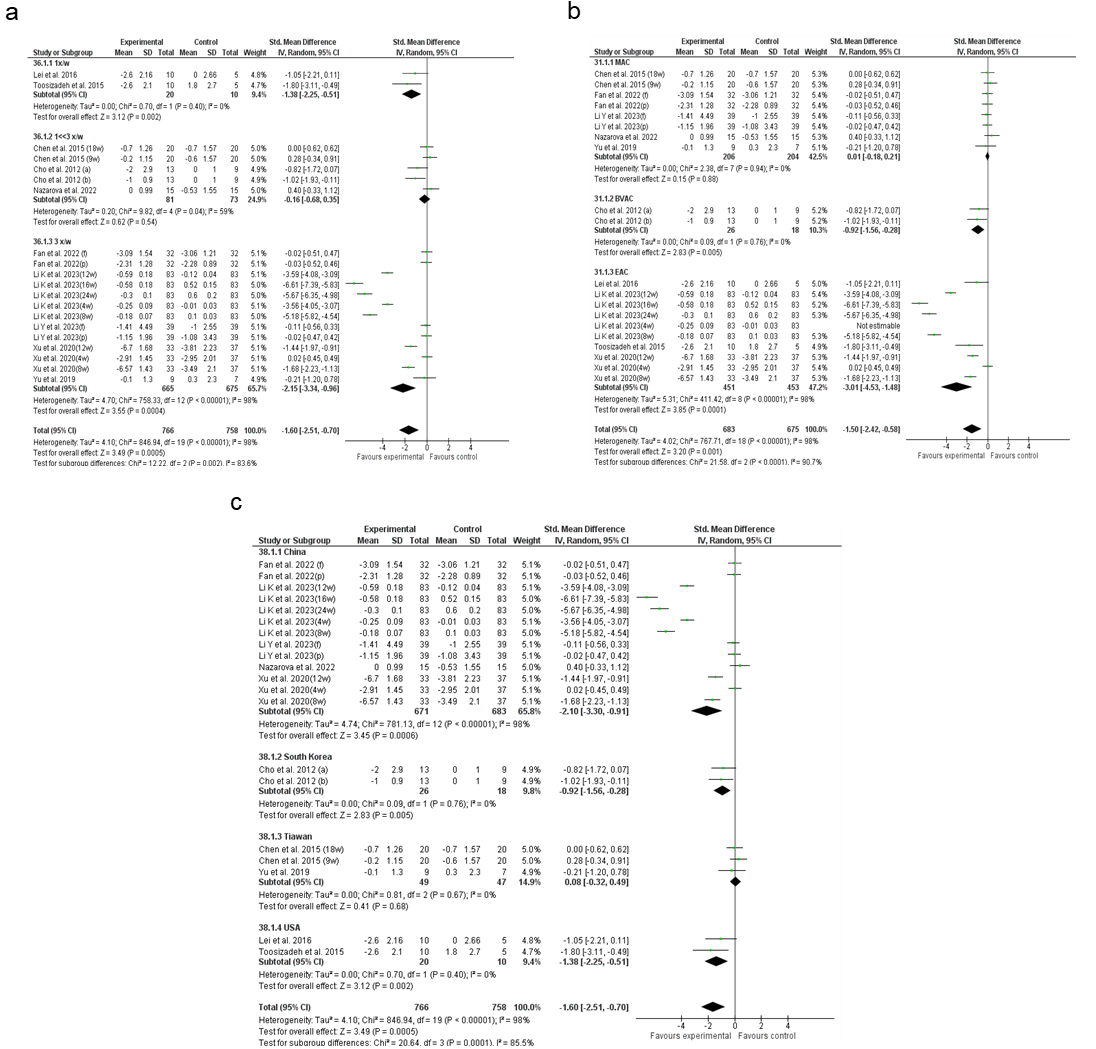


**Fig. S6** Subgroup analysis of UPDRS I based on a) session frequency, (b) acupuncture type and (c) country.

**Fig. S7** Subgroup analysis of UPDRS II based on a) needle specification, b) needling frequency, c) acupuncture areas and (d) session duration.

**Fig.** **S8** Subgroup analysis of UPDRS II based on a) session frequency, (b) acupuncture type and (c) country.

**Fig. S9** Subgroup analysis of UPDRS III based on a) needle specification, b) needling frequency, c) acupuncture areas and (d) session duration.

**Fig. S10** Subgroup analysis of UPDRS III based on a) session frequency, (b) acupuncture type and (c) country.

**Fig. S11** Subgroup analysis of UPDRS IV based on a) needle specification, b) needling frequency, c) acupuncture areas and (d) session duration.

**Fig. S12** Subgroup analysis of UPDRS IV based on a) session frequency, (b) acupuncture type and (c) country.

**Fig. S13** Meta-regression analysis showing the association between UPDRS IV with (a) needling frequency, (b) session duration, (c) session frequency, (d) acupuncture type and (e) country and the PDQ-39 with (f) acupuncture areas and (g) session frequency. Each bubble represents a study, with bubble size reflecting the precision of the estimate (inverse of within-study variance). The blue line indicates the regression trend.

**Fig. S14** Subgroup analysis of PDQ-39 based on a) needle specification, b) needling frequency, c) acupuncture areas and (d) session duration.

**Fig. S15** Subgroup analysis of PDQ-39 based on a) session frequency, (b) acupuncture type and (c) country.

**Fig. S16** Forest plot of the a) VAS, b) BD-II, c) HAM-D, d) HAM-A and e) Hoehn and Yahr stages. BD-II: bipolar disorder-II, HAM-D: Hamilton depression rating scale, HAM-A: Hamilton Anxiety Rating Scale.

**Fig. S17** Forest plot of the a) cadence, b) stride length, c) stride time, d) velocity, e) gait freezing and f) BBS. BBS: berg balance scale.


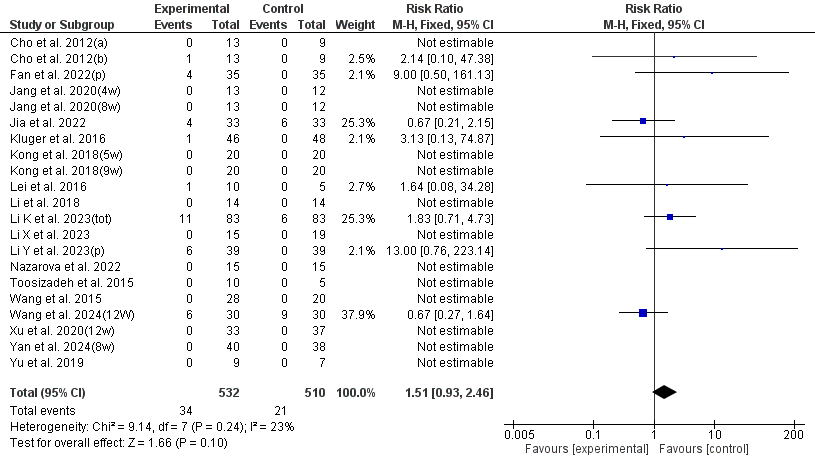
**Fig. S18** Forest plot of the relative risk of adverse events.

**Fig. S19** Sensitivity analysis of the a) UPDRS T, b) UPDRS I, c) UPDRS II, d) UPDRS III, e) UPDRS IV, f) PDQ-39 and g) VAS.
